# Cyclic Peptides Acting as Allosteric Inhibitors of Human Thymidylate Synthase and Cancer Cell Growth

**DOI:** 10.3390/molecules24193493

**Published:** 2019-09-26

**Authors:** Salvatore Pacifico, Matteo Santucci, Rosaria Luciani, Puneet Saxena, Pasquale Linciano, Glauco Ponterini, Angela Lauriola, Domenico D’Arca, Gaetano Marverti, Remo Guerrini, Maria Paola Costi

**Affiliations:** 1 Department of Chemical and Pharmaceutical Sciences, University of Ferrara, via Fossato di Mortara 17-19, 44100 Ferrara, Italy; salvatore.pacifico@unife.it; 2 Department of Life Sciences, University of Modena and Reggio Emilia, via Campi, 103, 41125 Modena, Italy; matteo.santucci86@gmail.com (M.S.); rosaria.luciani@libero.it (R.L.); puneet_saxena07@yahoo.com (P.S.); pasquale.linciano@unimore.it (P.L.); glauco.ponterini@unimore.it (G.P.); angela.lauriola@unimore.it (A.L.); 3 Department of Biomedical Sciences, Metabolic and Neural Sciences, University of Modena and Reggio Emilia, via Campi 287, 41125 Modena, Italy; domenico.darca@unimore.it (D.D.); gaetano.marverti@unimore.it (G.M.)

**Keywords:** cyclic peptides, enzyme inhibition, thymidylate synthase inhibitors, allosteric inhibitors, anticancer agents, ovarian cancer

## Abstract

Thymidylate synthase (TS) is a prominent drug target for different cancer types. However, the prolonged use of its classical inhibitors, substrate analogs that bind at the active site, leads to TS overexpression and drug resistance in the clinic. In the effort to identify anti-TS drugs with new modes of action and able to overcome platinum drug resistance in ovarian cancer, octapeptides with a new allosteric inhibition mechanism were identified as cancer cell growth inhibitors that do not cause TS overexpression. To improve the biological properties, 10 cyclic peptides (cPs) were designed from the lead peptides and synthesized. The cPs were screened for the ability to inhibit recombinant human thymidylate synthase (*h*TS), and peptide **7** was found to act as an allosteric inhibitor more potent than its parent open-chain peptide **[Pro^3^]LR**. In cytotoxicity studies on three human ovarian cancer cell lines, IGROV-1, A2780, and A2780/CP, peptide **5** and two other cPs, including **7**, showed IC_50_ values comparable with those of the reference drug 5-fluorouracil, of the open-chain peptide **[d-Gln^4^]LR**, and of another seven prolyl derivatives of the lead peptide **LR**. These promising results indicate cP **7** as a possible lead compound to be chemically modified with the aim of improving both allosteric TS inhibitory activity and anticancer effectiveness.

## 1. Introduction

Human thymidylate synthase (*h*TS) is a homodimeric protein involved in the folate metabolic pathway. It catalyzes the reductive methylation of 2′-deoxyuridine-5′-monophosphate (dUMP) to 2′-deoxythymidine-5′-monophosphate (dTMP) using the cofactor N^5^,N^10^-methylentetrahydrofolate (mTHF) [1]. It also regulates protein synthesis by interacting with its own mRNA as well as the mRNAs of other proteins involved in cell cycle and cell death [2,3,4,5]. *h*TS is largely expressed in different cancer types such as colorectal cancer, pancreatic cancer, and mesothelioma [6,7,8,9]. Thus, it is an important drug target for different cancer types. The clinically approved 5-fluorouracil (**5-FU**, the prodrug of 5-fluoro-2′-deoxyuridine-5′-monophosphate), raltitrexed (RTX) [10], and pemetrexed (PMX) [11] are TS-directed drugs acting as folate cofactor analogs. However, their prolonged use leads to *h*TS overexpression and results in drug resistance [12]. Our studies are focused on ovarian cancer, a severe pathology that affects 5% of women of all ages. The starting therapy relays on platinum drugs in combination with paclitaxel or doxorubicin. However, approximately 70% relapse due to insurgence of platinum drug resistance [13]. The resistant cancer type shows higher than normal levels of proteins involved in the replication pathways, including *h*TS, and cross-resistance with anti-*h*TS drugs [14,15,16] The above-mentioned anti-*h*TS drugs become ineffective. Therefore, we aimed at overcoming such a resistance mechanism by discovering new anti-TS drugs with modes of action different from those of the substrate or cofactor analogs.

**Figure 1:**
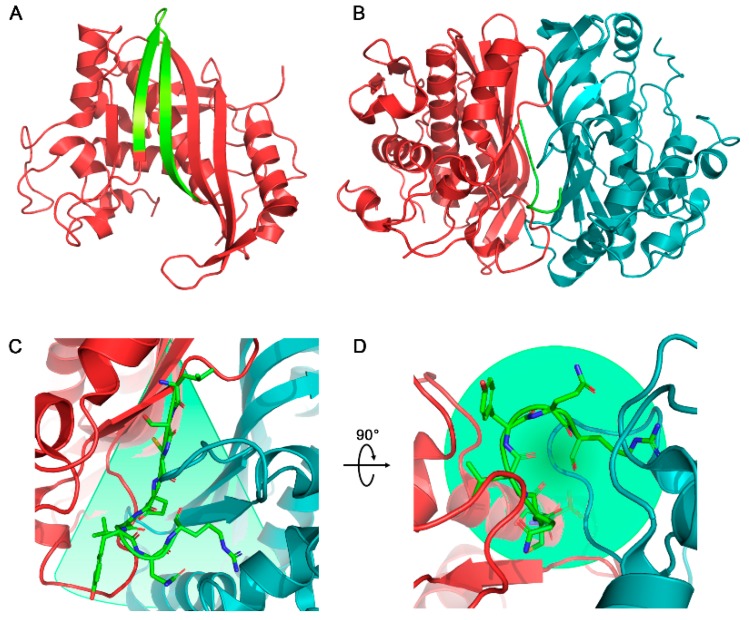
(**A**) Monomer of *h*TS (in red cartoon, PDB: 3N5E) with, highlighted in green, the region corresponding to the C20 peptide. (**B**) Crystal structure of the complex of *h*TS with the LR peptide (PDB: 3N5E). (**C**) Close-up on the cone-shaped binding cavity of the LR peptide in the interface between the two *h*TS monomers. (**D**) View from below of the cone-shape binding cavity, highlighting the helical-type conformations assumed by the LR peptide. The two monomers of *h*TS are represented in red and cyan cartoons; the LR peptide is represented in green ribbon.

To this aim, we previously identified some small peptidic inhibitors that mime a portion of the monomer–monomer interfacial region in the *h*TS dimer and bind therein [17,18,19,20,21]. More precisely, from a parent peptide, C20, whose sequence came from the interface region of *h*TS (Figure 1A), we synthesized a series of octapeptides. Two of them, **LR** (Leu^1^-Ser^2^-Cys^3^-Gln^4^-Leu^5^-Tyr^6^-Gln^7^-Arg^8^) and its more rigid diastereomer **[d-Gln^4^]LR**, proved to be allosteric inhibitors of the dimeric enzyme by stabilizing its di-inactive form, thus reducing the protein catalytic activity (Figure 1B) [17,21].

These peptides induced cancer cell growth inhibition in both platinum-sensitive and -resistant ovarian cancer cells. Fluorescence resonance energy transfer (FRET)-based target engagement experiments proved the binding of **LR** to TS in cells with good target specificity. In lysates of HEK293T cells, 2.5 micromoles/L of **LR** bound *h*TS, whereas only 30 nanomoles/L of the inhibitor interacted with other targets [19]. Proteomic experiments allowed the characterization of some proteins that are modulated by the peptides and that characterize the mechanism of action. We observed that the difference in binding sites between these peptides (allosteric) and such classical TS-active-site inhibitors as PMX and RTX goes together with a different protein set modulation by the drugs [22]. This also held true for the proline derivatives that we designed after the **LR** peptide (see below), a finding that points to a conserved mechanism of action in cells of **LR** and its proline mutants [20]. This protein set modulation was observed in different types of ovarian cancer cell models such as A2780, A2780/CP, IGROV-1, 2008, and C13* [17,20,21].

Closely connected with the intracellular activity is the low metabolic stability of the **LR** peptide [23]. Although a relatively fast proteolytic degradation did not prevent it from engaging the *h*TS target [19] and exhibiting an effect on cancer cell growth, we still expect higher activity from an increase in biostability. 

An important drawback in the design of peptidic inhibitors of protein–protein interactions is that peptides are often more conformationally disordered when excised from the stabilizing context of their protein interface domain. The larger the disorder, the lower the affinity (i.e., the higher the binding ΔG°) for the targets [17]. Thus, identifying a bioactive conformation with a low conformational disorder may be crucial to improving peptide activity. 

The crystal structure of the *h*TS/**LR** complex showed that the peptide binding site has the shape of an inverted cone (highlighted in green in Figure 1C,D), narrower at the top and broader at the bottom, where the **LR** peptide arranges with a longer extended portion that involves residues from Leu1 to Leu5 and a helical turn that involves the last three residues at the C-terminus. To improve the activity of the **LR** and **[d-Gln^4^]LR** octapeptides, we sequentially mutated residues 1 to 7 to proline, and examined the conformational and functional consequences of the introduction of this rigidifying residue in three regions, namely, near the C- and the N-termini and in the central portion of the peptides [20]. According to circular dichroism (CD) spectroscopic evidence, peptides **[Pro^1^]LR**, **[Pro^2^]LR**, and **[Pro^3^]LR** shared with **LR** the long-extended geometry of residues from 1 to 5 and similar tendencies to form compact helical turns that involve the residues near the C-terminus. Molecular dynamics simulations on **[Pro^2^]LR** showed that such a conformational preference resulted from intramolecular hydrogen bonds between CO(Gln4) and NH(Gln7) and between CO(Arg8) and, as donors, NH(Cys3) and NH(Gln4). On the other hand, mutation to proline at positions 5 and especially 6 and 7 generated peptides with lower propensities for assuming ordered secondary structures, with a distorted β-turn that included the Pro6 residue likely stabilized by a hydrogen bond between CO(Gln4) and NH(Gln7). Finally, consistently with the presence of such a rigidifying residue as proline at central positions, for **[Pro^4^]LR** and **[Pro^5^]LR** CD suggested a β-turn-like structure. Overall, the seven tested proline-mutated octapeptides showed conformational propensities similar to those of the starting peptide, **LR**, but more compact and ordered shapes. This structural feature, possibly combined with a stabilization versus peptidase degradation, may explain the stronger inhibition of ovarian cancer cell growth shown by **[Pro^3^]LR** and **[Pro^4^]LR** relative to the lead peptide, **LR** [20]. 

**Figure 2:**
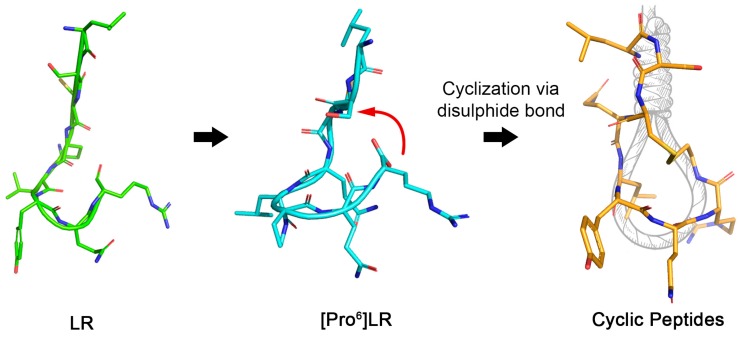
Design of cyclic peptides by introducing a disulfide bond based on the conformational studies previously performed on the linear peptide **LR**, and its restrained proline derivatives **[Pro^n^]LR**s.

Altogether, these observations on proline mutants confirm the value of these peptides as potential anticancer compounds. They also represent a starting point to develop new peptides that could enhance the positive features observed, in particular the lower structural flexibility and higher metabolic stability while maintaining the allosteric mechanism of *h*TS inhibition of the linear peptides, by binding at the protein interface. These features are expected to ensure a reduced *h*TS activity and also a reduced protein level even in platinum drug-resistant ovarian cancer cells. With this aim, we decided to move from linear peptides to cyclic peptides (cPs) that are known to show lower flexibility than the corresponding linear peptides and higher stability towards peptidase attack than the corresponding linear peptides [24,25]. In addition, peptide cyclization is known to improve such pharmacokinetic properties as membrane permeability and bioavailability [26] that are important general features for drug-like compounds. In the present work, we designed cPs involving 5–8 amino acid (AA) closures corresponding to the turn at the C-terminus of the **LR** peptide and an extended 0–2 AA chain at the N-terminus (see Figure 2). These cPs were synthesized, and their enzyme and cancer cell growth inhibition properties detected. Our work successfully allowed the identification of one important hit compound showing both *h*TS allosteric inhibition and cancer cell growth inhibition and other peptides with IC_50_ below 10 µM towards all ovarian cancer cells tested. 

## 2. Results and Discussion

### 2.1. Design of Cyclic Peptides

The design of 10 new cyclic derivatives was performed according to the conformational studies on **LR** (Leu^1^-Ser^2^-Cys^3^-Gln^4^-Leu^5^-Tyr^6^-Gln^7^-Arg^8^) and its more rigid d-Gln^4^ diastereomer, **[d-Gln^4^]LR**, and Pro^3^ derivative, **[Pro^3^]LR**. Since the C-terminus of these three peptides folds in a helical-type conformation occupying the basis of the conic binding cavity (Figure 1C,D), and in order to maintain the inhibition mechanism, we wanted to preserve this peculiar binding cavity for the new cyclic peptides giving them a noose-knot shape (Figure 2). Disulfide bonds are largely adopted for initial attempts to explore the efficacy of newly designed cPs [27]. The cPs’ delivery to the cancer cells is expected to happen using the peptides as such or by delivery through the SAINT-PhD system that we successfully adopted in our previous work with the linear peptides [17,18,19,20]. In this latest case, the cPs were complexed with the polymeric molecules of the delivery system, protected by protease attack and other chemical modifications up to when they were released intracellularly [17,21,23,28]. 

**Figure 3:**
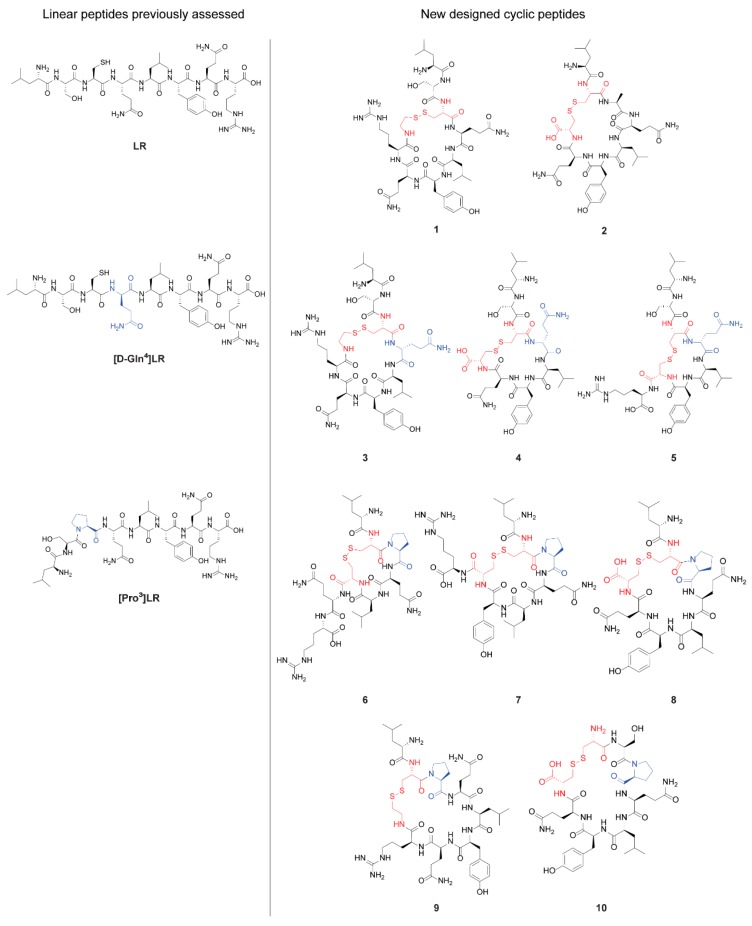
Chemical structures of the previously investigated peptides, **LR**, **[d-Gln^4^]LR**, and [**Pro^3^]LR,** and of the new cyclic peptides, **1**–**10**.

The cPs’ design aimed at exploring the functional consequences, in terms of both *h*TS inhibition and cytotoxicity, of such structural determinants as the relative sizes of the “linear” N-terminus-containing portion and of the C-terminus-containing cycle as well as the conformational rigidity of the latter. Peptides **1** and **2** (Figure 3) are the direct cyclic derivatives of the open-chain peptide **LR** with, respectively, six-amino-acid and seven-amino-acid cycles. In peptide **1** (Leu-Ser-c[Cys-Gln-Leu-Tyr-Gln-Arg-CAM]), the aminoacidic sequence of **LR** was left unaltered and the Cys^3^ was bound in a 6-term cycle with the Arg^8^ derivatized for this purpose at the C-terminus with a subunit of cysteamine (CAM). In contrast, in peptide **2** (Leu-c[Cys-Ala-Gln-Leu-Tyr-Gln-Cys]), in order to expand the cyclic peptide in a 7-term ring, an alanine was introduced in position 3 and the cycle was generated by connecting Cys^2^ with Cys^8^, which replaced the original Ser^2^ and Arg^8^, respectively (Figure 3). Peptides **3**–**5** derived from the cyclization of **[d-Gln^4^]LR** (Leu-Ser-Cys-d-Gln-Leu-Tyr-Gln-Arg). Peptide **3** (Leu-Ser-c[Cys-d-Gln-Leu-Tyr-Gln-Arg-CAM]) is the direct diastereomer of peptide **1** at Gln^4^. To evaluate the importance of the cycle dimension, in peptide **4** (Leu-Ser-c[Cys-d-Gln-Leu-Tyr-Gln-Cys]) a 6-term ring was obtained by replacing Arg^8^ of the linear peptide with Cys, leaving unaltered Cys^3^. In contrast, in peptide **5** (Leu-Ser-c[Cys-d-Gln-Leu-Tyr-Cys]-Arg), a constrained 5-term ring was generated by introducing a cysteine in position 7 and leaving unaltered the original terminal arginine residue (Figure 3). Starting from the partially constrained **[Pro^3^]LR** (Leu-Ser-Pro-Gln-Leu-Tyr-Gln-Arg), peptides **6**–**10** were designed and synthesized preserving Pro^3^. In peptide **6** (Leu-c[Cys-Pro-Gln-Leu-Cys]-Gln-Arg), the Ser^2^ was replaced with a cysteine and a 5-term cycle was generated with a residue of cysteine introduced at position 6 in the place of the original tyrosine. Moreover, in order to evaluate the influence of the ring size in these Pro^3^ derivatives, we explored the expansion of the ring into 6-term, 7-term, and 8-term cycles (peptides **7**, Leu-c[Cys-Pro-Gln-Leu-Tyr-Cys]-Arg, **8**, Leu-c[Cys-Pro-Gln-Leu-Tyr-Gln-Cys], and **9**, Leu-c[Cys-Pro-Gln-Leu-Tyr-Gln-Arg-CAM], respectively). To this end, the second residue of cysteine necessary to generate the disulfide bond with Cys^2^ was introduced to replace Gln^7^ of the peptide **[Pro^3^]LR** (peptide **7**) or the terminal Arg^8^ (peptide **8**). In peptide **9**, the Arg^8^ was derivatized for this purpose at the C-terminus with a subunit of CAM. Finally, in order to evaluate the importance in these cPs of the involvement of the N-terminus portion in the cycle, we generated the fully annular peptide **10** (c[Cys-Ser-Pro-Gln-Leu-Tyr-Gln-Cys]) by replacing both Leu^1^ and Arg^8^ with Cys (Figure 3).

### 2.2. Chemistry

**Scheme 1:**
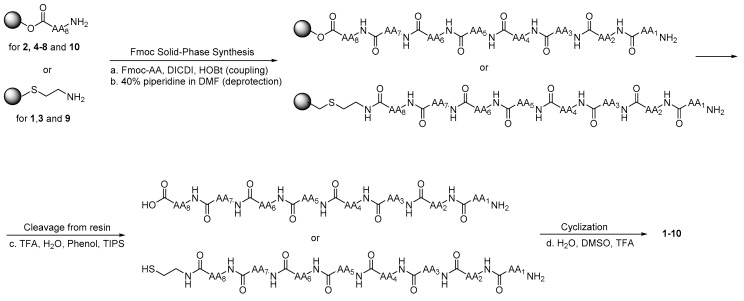
General procedure for the synthesis of the cyclic peptides **1**–**10**. Reagents and conditions: a) Fmoc-AA (4 eq.), DICDI (4 eq.), HOBt (4 eq.), DMF, r.t., 1 h; b) 40% piperidine in DMF, r.t., 1 h; c) TFA, H_2_O,phenol, TIPS 88:5:5:2 *v*/*v* 10 mL per 0.2 g of resin, r.t., 1.5 h; d) H_2_O, DMSO, TFA 75:25:0.1 *v*/*v*, r.t., 24 h.

The linear peptides were synthesized according to published methods [29] using Fmoc chemistry with a Syro XP multiple peptide synthesizer (MultiSynTech GmbH, Witten, Germany). Wang’s resin (for the synthesis of peptides **2**, **4**–**8**, and **10**) or (CAM)-2-chlorotrityl resin (for the synthesis of peptides **1**, **3,** and **9**) were used as the solid support [30]. The linear peptides were grown with deprotection/coupling cycles until the desired resin-bound peptide was completed. Coupling was performed using Fmoc-protected amino acids and diisopropylcarbodiimide (DIPCDI) and hydroxybenzotriazole (HOBt) as coupling reagents in dimethylformamide (DMF) at room temperature for 1.5 h. Deprotection was achieved with 40% of piperidine in DMF. The linear peptides were unbounded from the resin using a mixture of trifluoroacetic acid (TFA), water, phenol, and triisopropylsilane (TIPS) at room temperature for 1 h. Finally, the cyclization was performed solubilizing the linear purified analog in a mixture of H_2_O, DMSO, and TFA and reacted at room temperature for one day [31]. Both linear and cyclic peptides were purified by preparative HPLC (Scheme 1). 

### 2.3. Human Thymidylate Synthase Inhibitory Activity 

All synthesized cPs were screened for the ability to inhibit *h*TS and the results are reported in Table 1, where they are compared with the parent peptides, **LR**, **[d-Gln^4^]LR**, and **[Pro^3^]LR** [18,20]. All cPs showed higher water solubility than the parent linear peptides, and were first screened at 500 µM. Then, for the active ones, IC_50_ was measured reaching concentrations up to 3 mM. In the case of the linear peptides, we could only test the compounds up to 100 µM.

To track structure–activity relationships (SARs) and gain information about the relevant structural features that influence the inhibition potency, we analyzed the biological data as reported below. The derivatives of **LR** with a 6-AA cycle, cP **1** (Leu-Ser-c[Cys-Gln-Leu-Tyr-Gln-Arg-CAM]), and a 7-AA cycle, cP **2** (Leu-c[Cys-Ala-Gln-Leu-Tyr-Gln-Cys]), were 30- and 12-fold less active than **LR**, respectively, with IC_50_s versus *h*TS of 2125 and 900 µM. cPs **3** (Leu-Ser-c[Cys-d-Gln-Leu-Tyr-Gln-Arg-CAM]), **4** (Leu-Ser-c[Cys-d-Gln-Leu-Tyr-Gln-Cys]), and **5** (Leu-Ser-c[Cys-d-Gln-Leu-Tyr-Cys]-Arg), derived from the diastereomer **[d-Gln^4^]LR** with 6-, 6- and 5-AA ring closures were inactive at 500 µM. Because cP **3** is the analog of cP **1** with D-Gln^4^, contrary to what was found with the corresponding open-chain octapeptides, the D-chirality of Gln^4^, with the associated slightly larger rigidity, did not favor the anti-*h*TS activity of these 6-AA-ring cyclic peptides. Low, if any, inhibitory activities were also found with three out of the four cyclic analogs of **[Pro^3^]LR**, that is, with cPs expectedly featuring a narrower conformational freedom at Pro^3^, near the “knot”, relative to the corresponding Gln cyclic peptides. Peptides **6** (Leu-c[Cys-Pro-Gln-Leu-Cys]-Gln-Arg) and **9** (Leu-c[Cys-Pro-Gln-Leu-Tyr-Gln-Arg-CAM]), with 5- and 7-AA rings, respectively, were inactive at 500 µM both at room temperature and at 37 °C. On the other hand, a modest inhibitory activity (*h*TS IC_50_ of 856 µM) was detected for peptide **8** (Leu-c[Cys-Pro-Gln-Leu-Tyr-Gln-Cys]), the 7-AA-ring cP that differed from cP **2** for the Ala-to-Pro replacement at position 3. Given the similar activity between cP **2** and **8**, we must conclude that the Pro^3^ near the “knot” does not contribute to improving *h*TS inhibition of these 7-AA-ring cPs. Among the cPs derived from **[Pro^3^]LR**, peptide **7** (Leu-c[Cys-Pro-Gln-Leu-Tyr-Cys]-Arg), featuring a 6-AA ring, shows an inhibitory potency (*h*TS IC_50_ of 148 ± 40 µM) quite comparable with those of the lead peptides, **LR** and **[d-Gln^4^]LR** (Table 1). The kinetic inhibition pattern of cP **7** was thus further investigated and, in keeping with the inhibition behavior of the parent lead peptides [17,21], found to suggest an allosteric mechanism, with apparent values of K_I_ and K_I_′ similar to the IC_50_ value, namely, 120–150 µM (Figure S1, Supplementary Materials). 

Finally, the fully cyclic peptide **10** (c[Cys-Ser-Pro-Gln-Leu-Tyr-Gln-Cys]) showed an *h*TS IC_50_ of 484 µM, and was thus a slightly better inhibitor than the similar (except for the first two residues) 7-AA-ring cP **8** (Leu-c[Cys-Pro-Gln-Leu-Tyr-Gln-Cys]), and a slightly worse inhibitor than cP **7** (Leu-c[Cys-Pro-Gln-Leu-Tyr-Cys]-Arg) that differs from cP **10** in the size of the cycle and in the N- and C-terminus residues. 

In general, moving from the linear peptides to their cyclic analogs, the structural change is relevant. Indeed, as the data reported in Table 1 show, the types of cyclization we performed turned out to be rather detrimental for the inhibitory activity towards the *h*TS target. This suggests that the ring closure, even with the different features considered in the design, was complex and that the cPs could not fit properly into the cone-shaped interface binding site. Importantly, compound **7** shows an interesting allosteric inhibition of *h*TS.

cP **7** represents the most active compound stemming from the SAR analysis, and its inhibition is specific as demonstrated by the allosteric inhibition. On the other hand, it is clear that small changes in the structures reflect large changes in the overall structural profile and this causes a significant activity decrease. 

### 2.4. Cytotoxicity 

**Figure 4:**
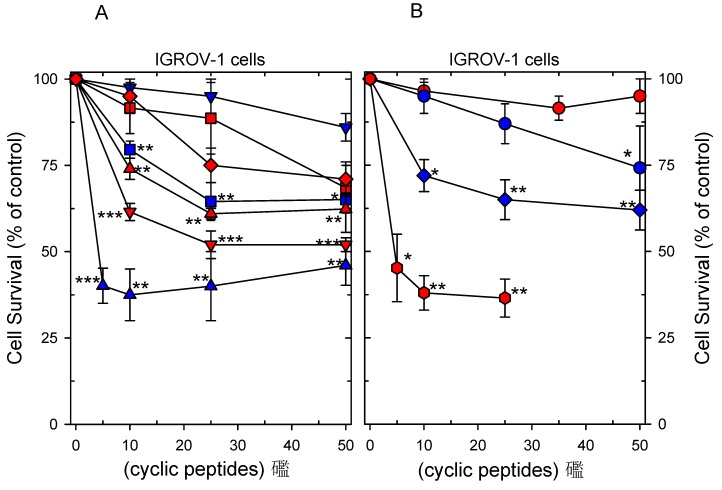
Dose–response curves of the effect of the cyclic peptides on cell growth of IGROV-1 cells. Panel **A**: **5** (blue triangles up), **3** (red triangles down), **9** (red triangles up), **7** (blue squares), **2** (red diamonds), **6** (red squares), **1** (blue triangles down). Panel **B**: **[d-Gln^4^]LR** (red hexagons), **10** (blue diamonds), **4** (blue circles), **8** (red circles). Data are mean values with standard deviation. Statistical significance by two-sided Student’s *t*-test and ANOVA followed by Tukey’s multiple comparison test, * *p* < 0.05; ** *p* < 0.01; *** *p* < 0.001.

**Figure 5:**
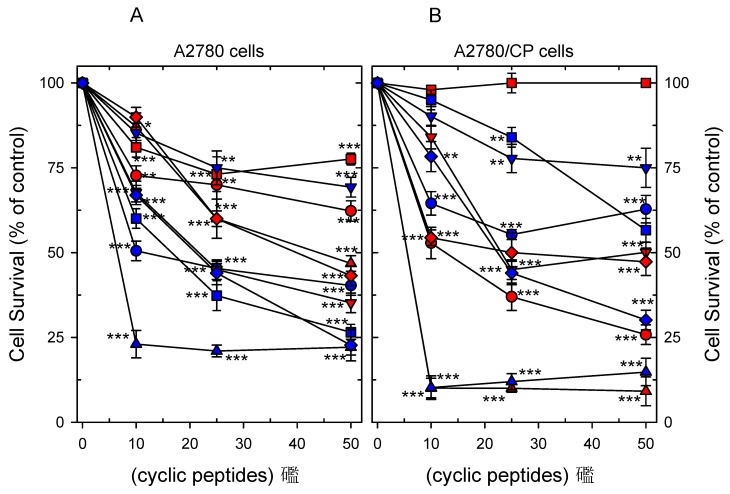
Dose–response curve of the effect of the cyclic peptides on the cell growth of A2780 (panel **A**) and A2780/CP (panel **B**) cell lines: **5** (blue triangles up), **9** (red triangles up), **4** (blue circles), **8** (red circles), **10** (blue diamonds)**, 7** (blue squares), **2** (red diamonds), **3** (red triangles down), **1** (blue triangles down), **6** (red squares). Data are mean values with standard deviation. Statistical significance by two-sided Student’s *t*-test and ANOVA followed by Tukey’s multiple comparison test, * *p* < 0.05; **; *p* < 0.01; *** *p* < 0.001.

**Figure 6:**
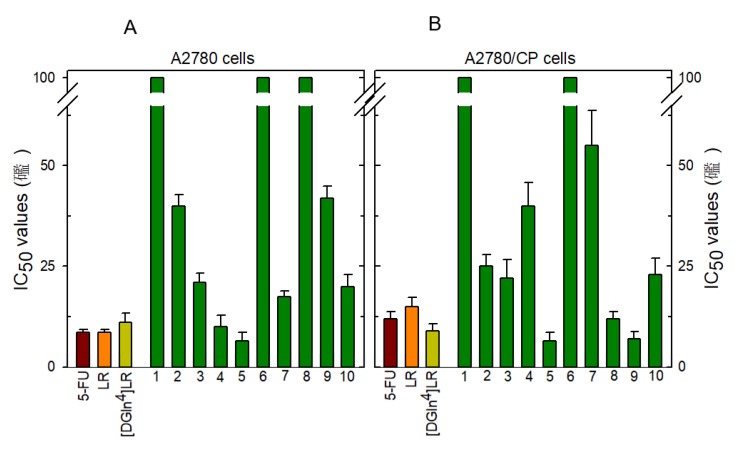
IC_50_ values for the inhibition of cell growth of IGROV-1 human ovarian cancer cells by the tested peptides. IC_50_ values of the 10 cyclic peptides (panel **A**, green bars) and of the 7 prolyl peptides (panel **B**, dark cyan bars) compared with **5-FU**, **LR**, and **[d-Gln4]LR**. Values are means ± SD of three separate experiments performed in duplicate.

**Figure 7:**
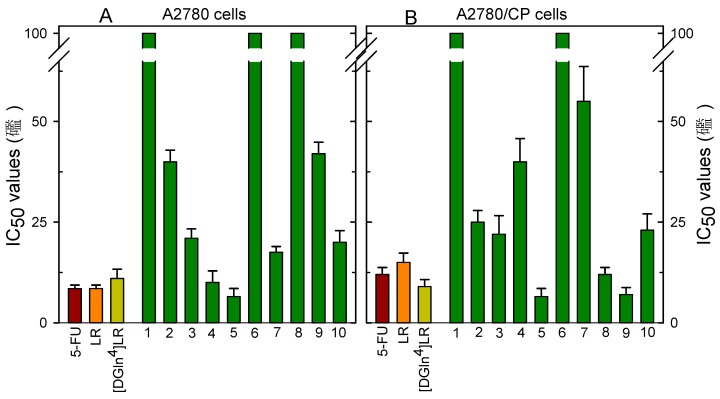
IC_50_ values of the cyclic peptides on cell growth of A2780 (**A**) and A2780/CP (**B**) human ovarian cancer cell lines. Values are means ± SD of three separate experiments performed in duplicate.

To evaluate the inhibition of cancer cell growth by the 10 cPs, we transfected them up to 50 µM into three ovarian cancer (OC) cell lines, namely, IGROV-1, A2780, and their cisplatin-resistant counterparts A2780/CP. These cell lines were chosen to compare the cytotoxicities of the cPs with those of the open-chain peptides **LR**, **[d-Gln^4^]LR**, and **[Pro^n^]LR** [17,20,21]. We selected A2780 and its platinum drug-resistant cell counterpart A2780/CP to observe if the new anti-*h*TS inhibitors could overcome platinum drug resistance. IGROV-1 is typically expressing higher than normal *h*TS levels, showing a lower sensitivity to **5-FU**, a prodrug inhibitor with a different biological mechanism compared to the peptides [20,22]. The three cell lines were considered a sufficient representative number of cancer cell models for our screening purposes. We first attempted to deliver the cPs directly to the cancer cells, but we could not observe any inhibitory effect (data not shown). For the transfection experiments, we employed the same peptide delivery system, SAINT-PhD, previously used in the cellular assays of the open-chain peptides [17,20,21]. Dose–response curves for all compounds are shown in Figure 4 and Figure 5, whereas the corresponding IC_50_ values are represented as vertical bars in Figure 6 and Figure 7. 

Quite at variance with the TS-inhibitory activities, peptide **5** (Leu-Ser-c[Cys-D-Gln-Leu-Tyr-Cys]-Arg) was the most effective among the 10 tested cPs against all three cell lines, with IC_50_ values lower than 10 µM, comparable with those of its open-chain parent, **[d-Gln^4^]LR**, and lower than **5-FU** (Figure 6A and Figure 7). cPs **3**, derived from **[d-Gln^4^]LR**, and **9**, **7**, and **10**, all derived from **[Pro^3^]LR**, showed intermediate cytotoxicities versus IGROV-1 cells at 10 and 25 µ M; cPs **2**, **6**, **1**, **4**, and **8** were the least active versus these cells. However, the A2780 and A2780/CP cell lines were much more sensitive to all of the 10 cPs except **6**, the peptide derived from **[Pro^3^]LR** with a 5-AA cycle (Figure 5B). As with the IGROV-1 cells, peptide **5** was the most potent one, together with peptide **9** (Leu-c[Cys-Pro-Gln-Leu-Tyr-Gln-Arg-CAM]) in the case of the cisplatin-resistant line. Compounds **4**, **5**, **8**, and **9** showed IC_50_ values lower than 10 µM and comparable with those of the reference drugs **5-FU**, **LR**, and **[d-Gln^4^]LR** for at least one of the cell lines. The other four peptides, **2**, **3**, **7**, and **10**, showed IC_50_ values below 25 µM versus at least one of the cell lines (Figure 7A,B). 

All tested compounds show a higher cytotoxic activity towards one or more cancer cell lines than *h*TS inhibition potency, thus supporting the idea that more targets and off-targets may be involved in the complex cellular environment. A role in increasing the cellular growth inhibition activity is quite certainly played by the increase in intracellular concentration of peptides relative to the extracellular concentration—for peptide **LR** with C13 cells, we found the intracellular concentration to be 15 times larger than the extracellular one when delivered through the SAINT-PhD system [23]. This ratio depends on both the thermodynamics of cellular internalization (i.e., the factors that affect the chemical potential of the peptide in the different compartments inside and outside cells) and the kinetics of peptide degradation, the latter being more relevant within cells than in the extracellular medium where they are protected from degradation by inclusion into the SAINT-PhD delivery system [23]. 

## 3. Conclusions

TS is one of the best-known anticancer targets. Its classical inhibitors bind at the substrate or cofactor binding pockets. However, upon prolonged treatment in the clinic, these inhibitors induce drug resistance mechanisms also related to TS overexpression. Ovarian cancer resistant to platinum drugs shows cross-resistance with classical *h*TS inhibitors; therefore, treatments with these drugs cannot circumvent the resistance problem. We previously identified some octapeptides that allosterically inhibited *h*TS without causing its cellular overexpression. Among them, peptide **LR** and its diastereomer **[d-Gln^4^]LR** were the most interesting ones. The X-ray crystallographic structure of the *h*TS/**LR** complex provides conformational details on the binding mode of **LR** at the protein interface, showing that it occupies a conic space region. The occurrence of an allosteric inhibition was confirmed by the inhibition pattern. To produce more potent inhibitors by decreasing the conformational disorder of these peptides, we first mildly rigidified the open-chain **LR** peptide by introducing a proline residue along its sequence and then designed 10 cyclic peptides from either **LR**, **[d-Gln^4^]LR**, or **[Pro^3^]LR**. Most of the synthesized peptides preserved one or the other of the residues that contributed to increase the TS inhibitory activity and cancer cell growth inhibition, namely, D-Gln^4^ and Pro^3^. 

Peptides **7** (Leu-c[Cys-Pro-Gln-Leu-Tyr-Cys]-Arg), **8** (Leu-c[Cys-Pro-Gln-Leu-Tyr-Gln-Cys]), and **10** (c[Cys-Ser-Pro-Gln-Leu-Tyr-Gln-Cys]) are the three best *h*TS inhibitors among the 10 tested cPs. All of them feature, at position 3, a proline residue, which, in contrast with the D-Gln^4^ residue, can rigidify the conformations of the three cyclic peptides in a way that is useful for inhibiting *h*TS. Their cycles include, in order of increasing anti-*h*TS activity, 6 (cP **7**), 8 (cP **10**), and 7 AA (cP **8**). The cycle size, therefore, is not a strong structural predictor of the inhibitory activity, provided it is larger than 5 AA (non-inhibiting cPs **5** and **6**). Complementarily, an extended N-terminus portion in these noose-knot peptides, here either absent (cP **10**) or consisting in only one residue (cPs **7** and **8**), does not seem to be relevant for their abilities to inhibit *h*TS. 

Of the two cPs with a 5-AA ring, peptide **6**, inactive versus *h*TS, was also almost non-cytotoxic towards the three cell lines employed. On the other hand, peptide **5**, also inactive as an *h*TS inhibitor at the tested concentrations, was the most cytotoxic among the 10 cPs. 

The differences between the *h*TS inhibitory activities and the cytotoxicities are due to many factors that may influence the activity of a drug during the different steps of the membrane penetration process and target engagement. The IC_50_s determined from cell growth inhibition were systematically lower, or much lower, than those associated with *h*TS inhibition. The characterization of the cellular mechanism of action is however beyond the scope of this preliminary note. The efficiencies of all mentioned factors that may influence the cPs’ intracellular activity are likely determined by structural descriptors of the cyclic peptides that are different from those that control their affinities towards recombinant *h*TS. For example, different cyclic peptides may accommodate differently into the delivery system vesicles, thus yielding SAINT-PhD/cPs complexes with different chemical potentials that are completely unrelated to the affinities of the cPs for *h*TS. Or, again, as is the case with the **[Pro^n^]LR** relative to the **LR** peptides [20,21,22], different cyclic peptides may be differently prone to intracellular degradation by peptidases and, hence, the observed lack of quantitative correlation between the *h*TS inhibitory activities and the cytotoxicities. 

While these results stimulate many questions concerning the efficiency of cellular internalization and intracellular stability and the intracellular mechanism of action of the cPs, questions that are being addressed in scheduled experiments, our first screening of these 10 cPs leaves us with a cP, compound **5**, that was active against all cancer cells with IC_50_s lower than 10 µ M, and a cP, compound **7**, that inhibited cancer cell growth and was by far the best *h*TS inhibitor. Thus, while compound **5** may give us hints about the structural requirements necessary to achieve good activity towards cancer cells, compound **7** can be considered a starting lead useful for the further chemical optimization of *h*TS-inhibiting cyclic peptides. 

## 4. Materials and Methods

### 4.1. Synthesis and Purification

#### 4.1.1. Linear Peptides

Linear **LR** analogs were synthesized using a Syro XP multiple peptide synthesizer (MultiSynTech GmbH, Witten, Germany). Chemicals and solvents were purchased from Sigma Aldrich (St. Luis, MO, United States). Wang resin preloaded with the C-terminal amino acid or (CAM)-2-chlorotrityl resin were used as a solid support for the synthesis of the derivatives. Fmoc-amino acids (4 equivalents) were sequentially coupled to the growing peptide chain using DIPCDI and HOBt (N,N’-diisopropylcarbodiimide/1-hydroxybenzotriazole) (4 equivalents) as activating agents at room temperature for 1 h. Deprotection of the Fmoc-growing peptide was performed with 40% piperidine in DMF followed by the coupling with the subsequent amino acids. Deprotection and coupling cycles were repeated until the desired peptide-bound resin was completed. The protected peptide-resin was treated with reagent B (trifluoroacetic acid (TFA), H_2_O, phenol, and TIPS 88:5:5:2 *v*/*v*; 10 mL per 0.2 g of resin) [30] at room temperature for 1.5 h. After filtration of the resin, the solvent was concentrated in vacuo and the residue triturated with ethyl ether. Crude peptides were purified by preparative reversed-phase HPLC using a Water Delta Prep 3000 system with a Jupiter column C18 (250 × 30 mm, 300 A, 15 µm spherical particle size, Waters Corporation, Milford, MA, United States). The column was perfused at a flow rate of 20 mL/min with a mobile phase containing solvent A (5%, *v*/*v*, acetonitrile in 0.1% TFA) and a linear gradient from 0 to 50% of solvent B (60%, *v*/*v*, acetonitrile in 0.1% TFA) over 25 min for the elution of peptides. Analytical HPLC analyses were performed on a Beckman 116 liquid chromatograph equipped with a Beckman 166 diode array detector (Beckman Coulter, Brea, CA, United States). Analytical purity of the peptides was assessed using a Zorbax C18 column (4.6 × 150 mm, 3 µm particle size, Agilent, Santa Clara, CA, United States) with the above solvent system (solvents A and B) programmed at a flow rate of 0.7 mL/min using a linear gradient from 0% to 100% B over 25 min. All analogs showed ≥95% purity when monitored at 220 nm. Molecular weight of final compounds was determined by a mass spectrometer ESI Micromass ZMD-2000 (Waters Corporation, Milford, MA, United States) and similar to the MS spectra already reported [21].

#### 4.1.2. Cyclic Peptides (**1**–**10**)

To carry out and assess peptide cyclization, we followed a chemical procedure previously adopted by some of us for the synthesis of cyclic urotensin II analogs [31]. In short, the respective linear purified peptides were dissolved in a mixture of H_2_O, DMSO, and TFA (75:25:0.1 *v*/*v*) at the concentration of 1mg/mL and stirred at room temperature for 24 h. We monitored the reaction by analytical HPLC, mass spectrometry, and the Ellman test, being careful to prevent the final Ellman’s test solution to become acidic [32]. After partial evaporation of the solvent, the product was purified by preparative HPLC as mentioned above to yield the desired cyclic compound (Supplementary Materials).

Leu-Ser-c[Cys-Gln-Leu-Tyr-Gln-Arg-CAM-] (**1**)
15% yield. Retention time (Rt): 8.63 min. ESI-MS [M + H]^+^ calcd: 1067.513; found: 1068.006.
Leu-c[Cys-Ala-Gln-Leu-Tyr-Gln-Cys] (**2**)
30% yield. Rt: 11.08 min. ESI-MS [M + H]^+^ calcd: 939.407; found: 939.852.
Leu-Ser-c[Cys-D-Gln-Leu-Tyr-Gln-Arg-CAM-] (**3**)
13% yield. Rt: 8.66 min. ESI-MS [M + H]^+^ calcd: 1067.513; found: 1067.974.
Leu-Ser-c[Cys-D-Gln-Leu-Tyr-Gln-Cys-] (**4**)
18% yield. Rt: 12.47 min. ESI-MS [M + H]^+^ calcd: 955.402; found: 955.402.
Leu-Ser-c[Cys-D-Gln-Leu-Tyr-Cys-]Arg (**5**)
29% yield. Rt: 10.74 min. ESI-MS [M + H]^+^ calcd: 983.444; found: 983.904.
Leu-c[Cys-Pro-Gln-Leu-Cys-]Gln-Arg (**6**)
25% yield. Rt: 8.42 min. ESI-MS [M + H]^+^ calcd: 958.460; found: 958.756.
Leu-c[Cys-Pro-Gln-Leu-Tyr-Cys-]Arg (**7**)
18% yield. Rt: 9.33 min. ESI-MS [M + H]^+^ calcd: 993.465; found: 993.561.
Leu-c[Cys-Pro-Gln-Leu-Tyr-Gln-Cys-] (**8**)
22% yield. Rt: 12.47 min. ESI-MS [M + H]^+^ calcd: 965.422; found: 966.074.
Leu-c[Cys-Pro-Gln-Leu-Tyr-Gln-Arg-CAM] (**9)**
10% yield. Rt: 9.11 min. ESI-MS [M + H]^+^ calcd: 1078.331; found: 1078.251.
c[Cys-Ser-Pro-Gln-Leu-Tyr-Gln-Cys] (**10**)
25% yield. Rt: 9.23 min. ESI-MS [M + H]^+^ calcd: 939.370; found: 939.730.

### 4.2. Thymidylate Synthase Purification, Characterization, and Inhibition

*h*TS was purified as previously reported [21]. The enzyme solution was thawed the day of the experiment and the enzyme concentration was determined by UV−Vis spectroscopy (Varian Cary 100, Agilent, Santa Clara, CA, USA) using ε_280_ = 89000 M^−1^ cm^−1^ and Mr = 74 229. The thawed protein solution was kept constantly at 4 °C. In this condition, the enzyme was able to reproduce normal kinetic activity values (K_M_ dUMP = 10−12 μM, K_M_ mTHF = 4−6 μM, k_cat_ = 0.8−0.9 s^−1^). The peptide solution was mixed or vortexed in order to help solubilization; the precise concentration was determined through UV−Vis spectroscopy. A stock solution of mTHF was prepared in carbonate buffer. The stock was prepared in the range of 5 to 6 mM. A stock solution of dUMP was prepared in bidistilled water in the range of 10−12 mM. Peptide inhibitors were tested without incubation with *h*TS in phosphate buffer, pH 6.9, at 25 °C. A 1 mL amount of assay buffer consisted of 50 mM TES, pH 7.4, containing 25 mM MgCl_2_, 6.5 mM HCHO, 1 mM EDTA, 75 mM β-mercaptoethanol (β-ME), 0.120 mM dUMP, and 0.060 mM mTHF. Following the addition of the enzyme (300 nM) and inhibitor mixture, the absorbance was monitored at 340 nm in a UV−Vis spectrophotometer for 3 min. In the control sample, an equal aliquot of enzyme alone was added. *h*TS activity inhibition percentages (I%) values were measured at 0.100−3000 µM concentration of inhibitor. The concentration of the peptide that showed the 50% activity inhibition was measured for the most active peptides. The kinetic inhibition pattern was studied for compound **7** [12]. The inhibition values are expressed as mean ± SEM; *n* = 3.

### 4.3. Cell Lines

The IGROV-1, A2780, and A2780/CP human ovarian cancer cell lines [33] were grown as monolayer in RPMI 1640 medium containing 10% heat-inactivated fetal bovine serum and 50 µg/mL gentamycin sulfate (Euroclone, Devon, UK). Cultures were equilibrated with humidified 5% CO_2_ in air at 37 °C. The cDDP-resistant variant A2780/CP cell line, derived from the parent A2780 cell line, is about 10-fold resistant to cDDP and was developed by monthly exposure to cDDP, followed by chronic exposure to stepwise increases in cDDP concentration. All studies were performed in *Mycoplasma* negative cells, as routinely determined with the MycoAlert Mycoplasma detection kit (Lonza, Walkersville, MD, USA).

### 4.4. Treatment with Peptides in Presence of SAINT-PhD Delivery System

Treatment with each peptide was performed according to the standard transfection protocol of the SAINT-PhD delivery system (Synvolux Therapeutics, Leiden, Netherlands) [17,21,23,28,34]. Complexes of peptide (µg) with SAINT-PhD (µL) were prepared at appropriate ratios corresponding to the approximate µM concentrations indicated. For each protein sample, complexes were prepared as follows: for the treatment of a 24-well plate, the appropriate amount of each peptide was diluted in HBS, then SAINT-PhD was pipetted into the solution without vortexing; then, the mixture was incubated for 5 min at room temperature, and filled up to 500 µL with serum-free medium. The culture medium was aspirated from the cells, the SAINT-PhD/peptide complex was added to the wells and incubated 4 h (37 °C, 5% CO_2_). Afterward, complete RPMI was added to reach the appropriate volume for maintaining the cell culture for the duration of the experiments.

### 4.5. Cytotoxicity Assay of Peptides

Cell growth was determined using a modified crystal violet assay [35]. On selected days, the tissue culture medium was removed, and the cell monolayer fixed with methanol and stained with 0.2% crystal violet solution in 20% methanol for at least 30 min. After being washed several times with distilled water to remove excess dye, the cells were left to dry. The incorporated dye was solubilized in acidified isopropanol (1N HCl: 2-propanol, 1:10). After appropriate dilution, absorbance was determined spectrophotometrically at 540 nm. The extracted dye was proportional to cell biomass. The percentage of cytotoxicity was calculated by comparing the absorbance of cultures exposed to the drug to unexposed (control) cultures.

### 4.6. Statistical Analysis

Data indicate mean values and standard deviation. In addition, *p*-values were calculated with the two-sided Student’s *t*-test and ANOVA followed by Tukey’s multiple comparison test. * *p* < 0.05, ** *p* < 0.01; *** *p* < 0.001.

## Figures and Tables

**Table 1:** *h*TS inhibitory activity of the previously assessed peptides **LR**, **[d-Gln^4^]LR**, and [**Pro^3^]LR** and the new cyclic peptides **1**–**10**. The assay was performed under stirring to prevent precipitation of the peptides and at room temperature (20–22 °C). To improve solubility, peptides inactive at room temperature were also tested at 37 °C.

Compound	Primary Sequence	% in *h*TS ^a^ (IC_50_ in µM) ^a^
**LR**	Leu-Ser-Cys-Gln-Leu-Tyr-Gln-Arg	(75) ^b^
**[d-Gln^4^]LR**	Leu-Ser-Cys-d-Gln-Leu-Tyr-Gln-Arg	(68) ^b^
**[Pro^3^]LR**	Leu-Ser-Pro-Gln-Leu-Tyr-Gln-Arg	25% at 100 µM ^c^
**1**	Leu-Ser-c[Cys-Gln-Leu-Tyr-Gln-Arg-CAM]	25% at 400 µM and r.t. under stirring (2125)
**2**	Leu-c[Cys-Ala-Gln-Leu-Tyr-Gln-Cys]	36% at 400 µM and r.t. under stirring (900)
**3**	Leu-Ser-c[Cys-d-Gln-Leu-Tyr-Gln-Arg-CAM]	N.I. at 500 µM and r.t. N.I. at 500 µM and 37 °C under stirring
**4**	Leu-Ser-c[Cys-d-Gln-Leu-Tyr-Gln-Cys]	N.I. at 500 µM and r.t. N.I. at 500 µM and 37 °C under stirring
**5**	Leu-Ser-c[Cys-d-Gln-Leu-Tyr-Cys]-Arg	N.I. at 500 µM and r.t. N.I. at 500 µM and 37 °C under stirring
**6**	Leu-c[Cys-Pro-Gln-Leu-Cys]-Gln-Arg	N.I. at 500 µM and r.t. N.I. at 500 µM and 37 °C under stirring
**7**	Leu-c[Cys-Pro-Gln-Leu-Tyr-Cys]-Arg	76% at 500 µM, 59% at 100 µM and r.t. under stirring (148) Allosteric inhibition profile (see Figure S1)
**8**	Leu-c[Cys-Pro-Gln-Leu-Tyr-Gln-Cys]	38% at 500 µM and r.t. under stirring (856)
**9**	Leu-c[Cys-Pro-Gln-Leu-Tyr-Gln-Arg-CAM]	N.I. at 500 µM and r.t. N.I. at 500 µM and 37 °C under stirring
**10**	c[Cys-Ser-Pro-Gln-Leu-Tyr-Gln-Cys]	50% at 500 µM and r.t. under stirring (474)

N.I., No inhibition; r.t., room temperature. ^a^ standard error is ±20% of the value; ^b^ Ref. [18]; ^c^ Ref. [20].

## Supplementary Materials

The following supplementary material are available online. Figure S1: Allosteric inhibition profile for peptide **7**; Mass spectra and analytical HPLC chromatograms of the final cyclic peptides.
