# Supplementary material for: Cyclic Peptides Acting as Allosteric Inhibitors of Human Thymidylate Synthase and Cancer Cell Growth
Source: Molecules. 2019 Sep 26;24(19):3493. doi: 10.3390/molecules24193493 (PMC6804113; doi:10.3390/molecules24193493)

Cyclic peptides acting as allosteric inhibitors of human thymidylate synthase and cancer cell growth.

Salvatore Pacifico,[1] Matteo Santucci,[2] Rosaria Luciani,[2] Puneet Saxena[2], Pasquale Linciano,[2] Glauco Ponterini,[2] Domenico D'Arca,[3] Gaetano Marverti,[3] Remo Guerrini,[1] M. Paola Costi[2]

[1] *Department of Chemical and Pharmaceutical Sciences, University of Ferrara, via Fossato di Mortara 17-19, 44100 Ferrara, Italy.*
[2] *Department of Life Sciences, University of Modena and Reggio Emilia, via Campi, 103, 41125 Modena, Italy.*
[3] *Department of Biomedical Sciences, Metabolic and Neural Sciences, University of Modena and Reggio Emilia, via Campi 287, 41125 Modena, Italy.*

TABLE OF CONTENT

Figure SI-1. Allosteric inhibition profile for peptide **7.** SI-2

Mass spectra and analytical HPLC chromatograms of the final cyclic peptides. SI-3

Figure SI-1. Allosteric inhibition profile for peptide **7**; V= initial rate measured as the change in absorbance at 340 nm per minute; [S] = [MTHF]/μM.

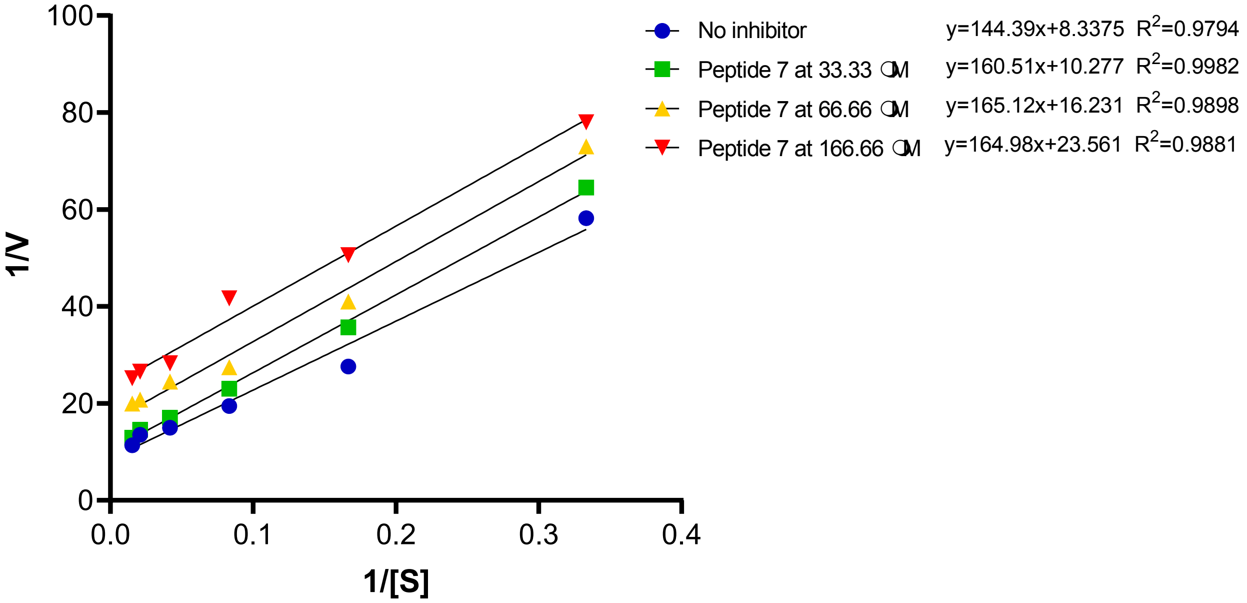

Mass spectra and analytical HPLC chromatograms of the final cyclic peptides.

Leu-Ser-c[Cys-Gln-Leu-Tyr-Gln-Arg-CAM] (1)

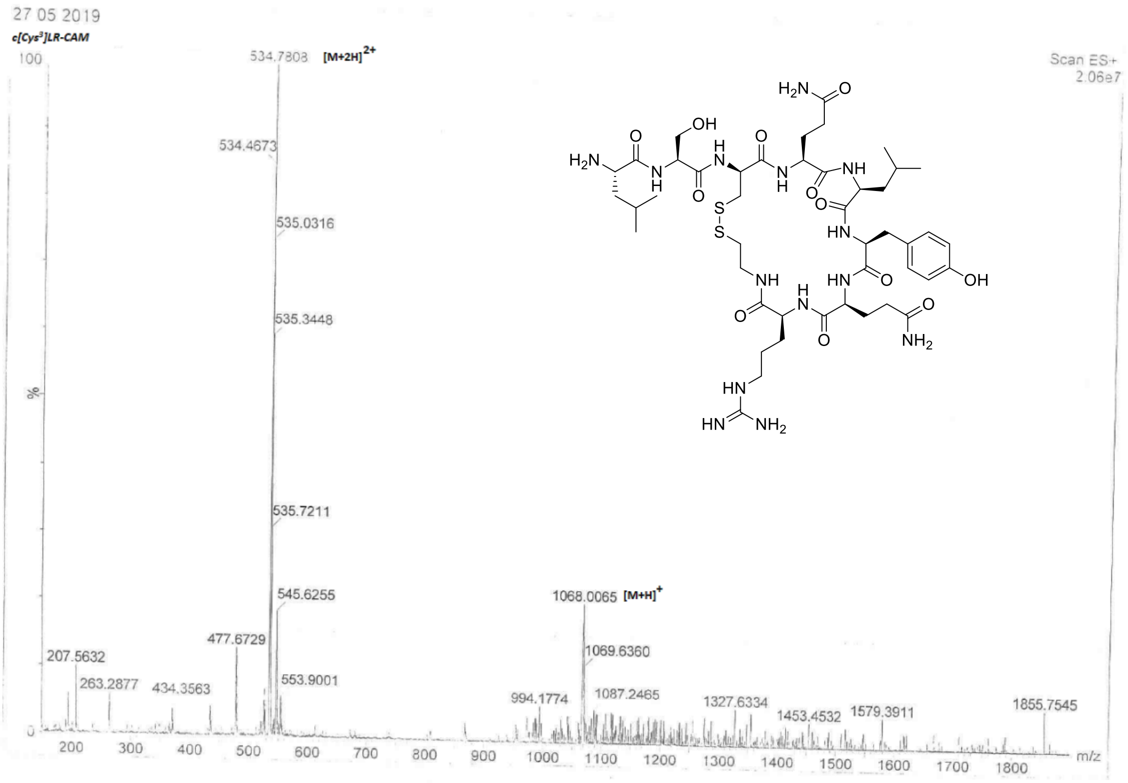

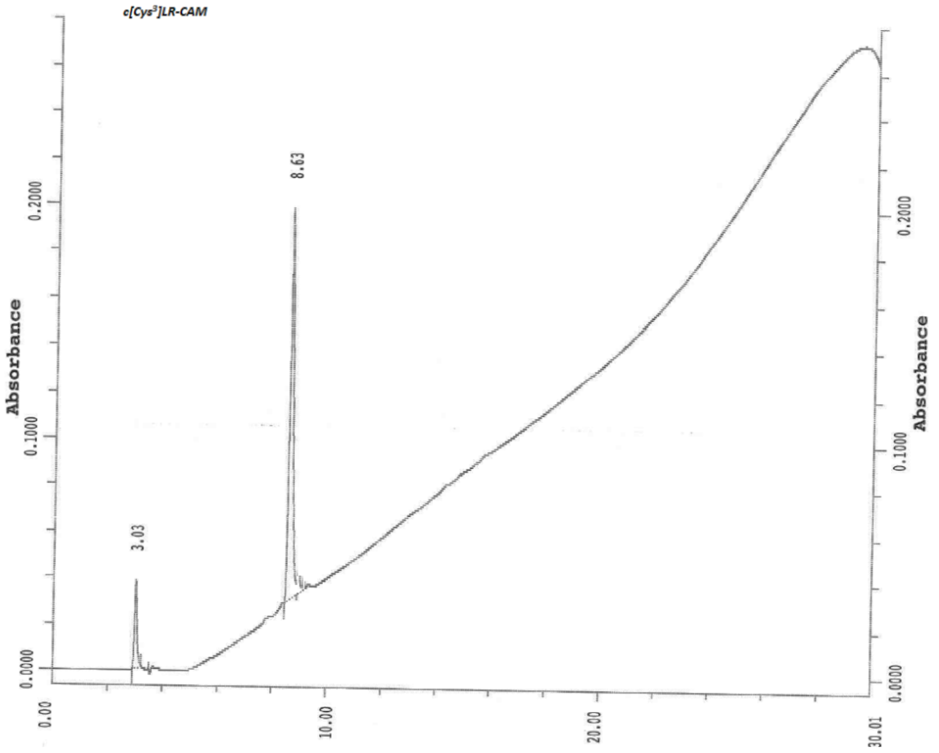

Leu-c[Cys-Ala-Gln-Leu-Tyr-Gln-Cys] (2)

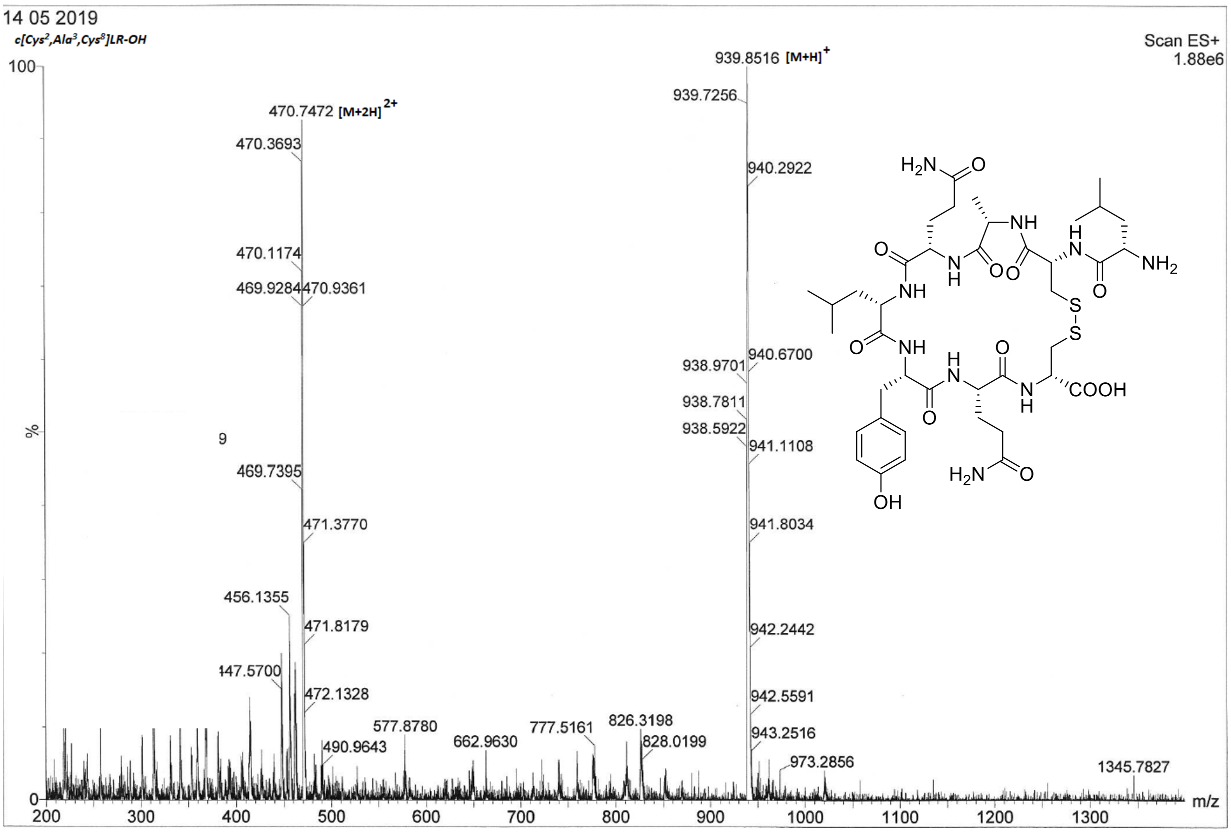

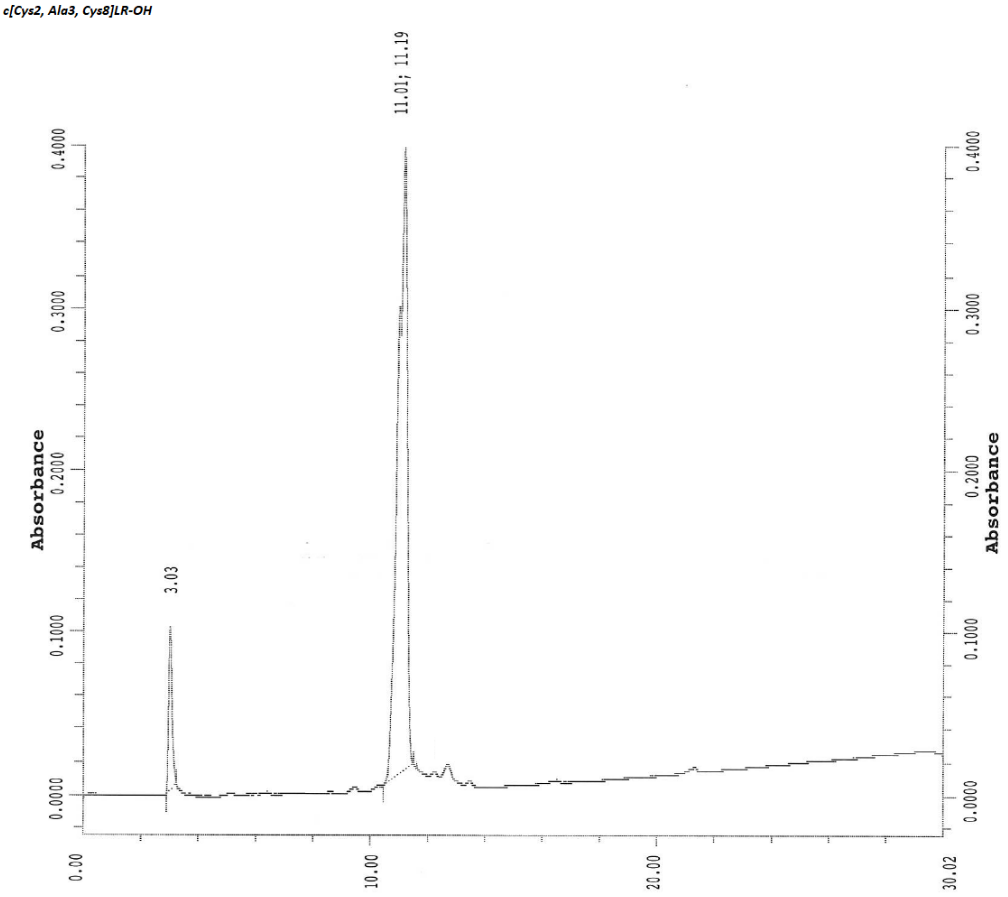

Leu-Ser-c[Cys- D-Gln-Leu-Tyr-Gln-Arg-CAM] (3)

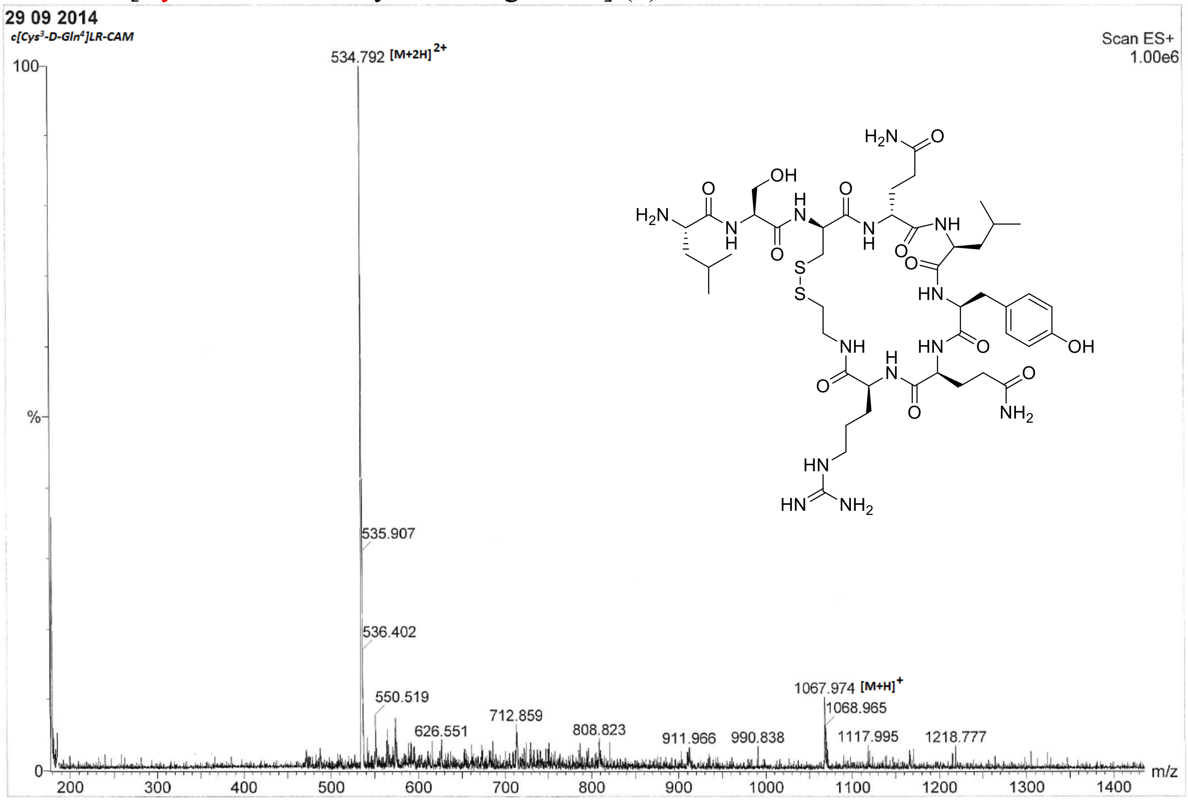

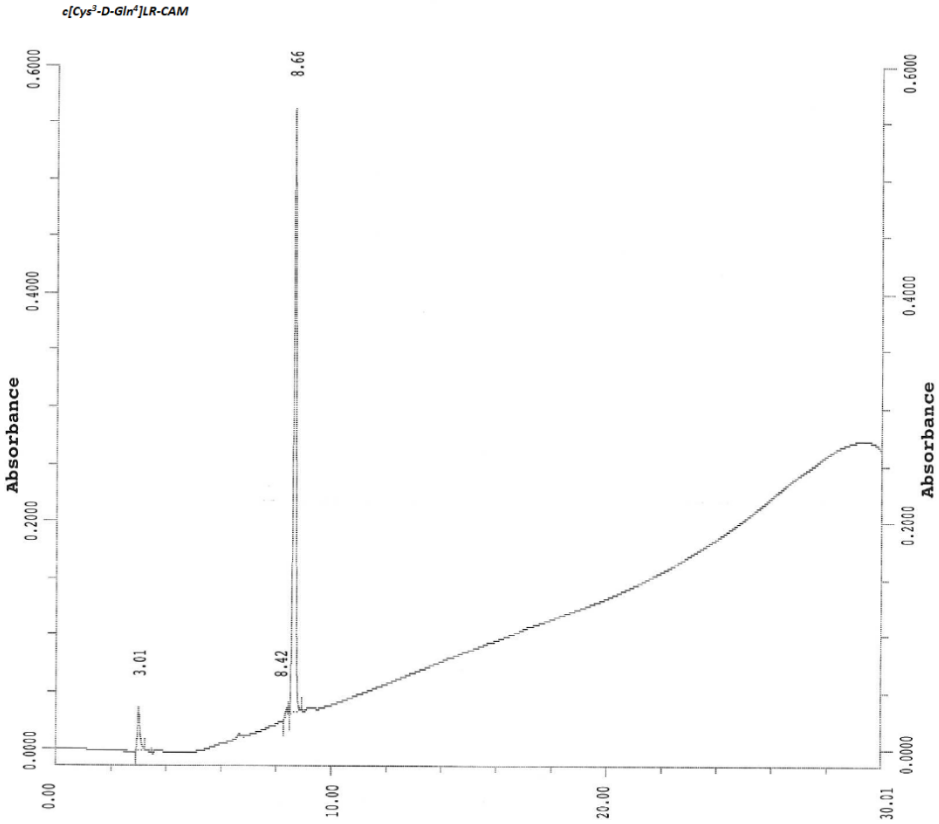

Leu-Ser-c[Cys- D-Gln-Leu-Tyr-Gln-Cys] (4)

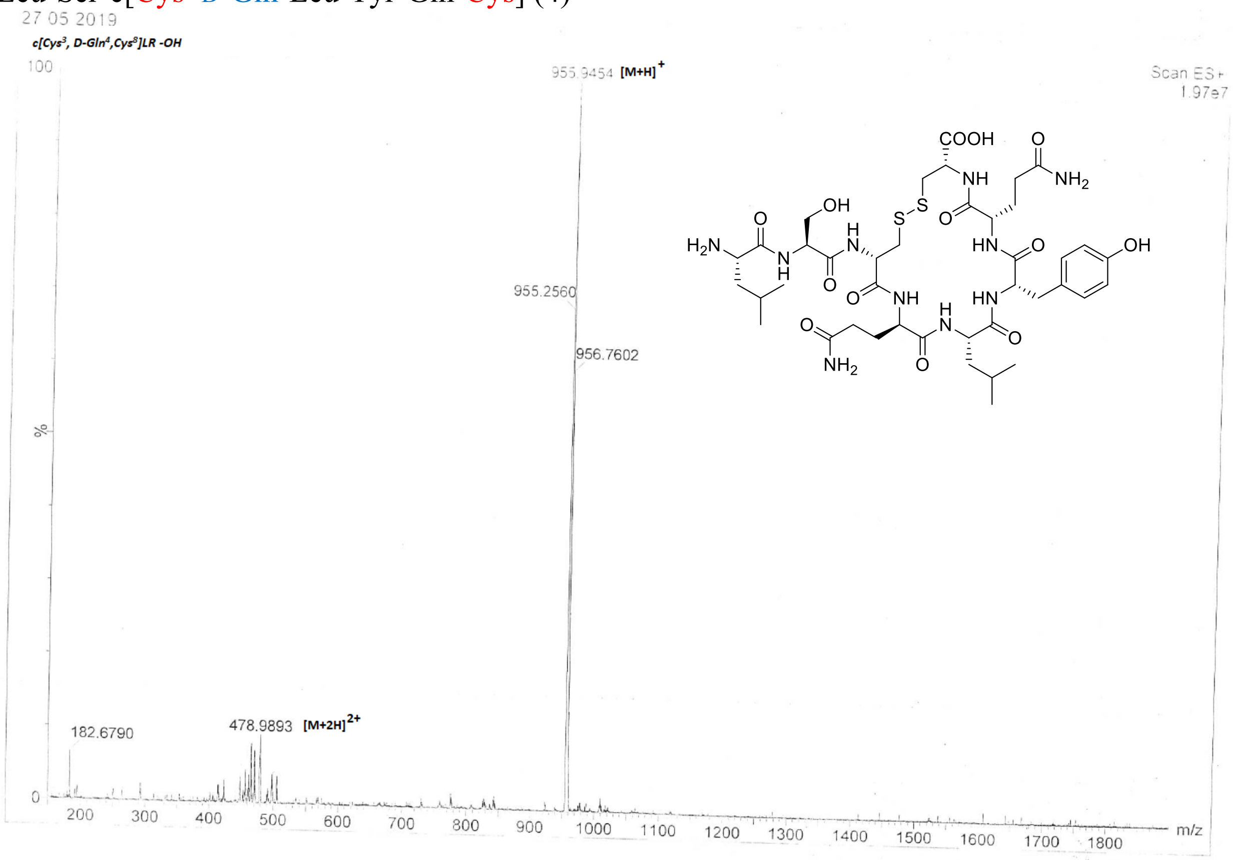

c[Cys2, Pro3, Cys8]LR-OH

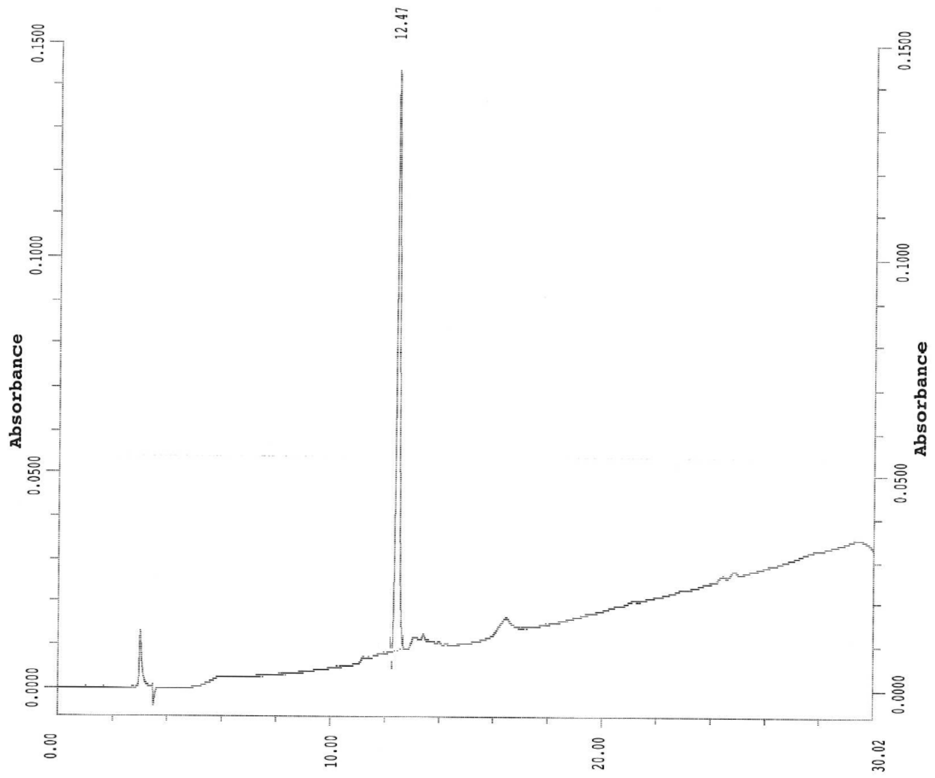

Leu-Ser-c[Cys- D-Gln-Leu-Tyr-Cys-]Arg (5)

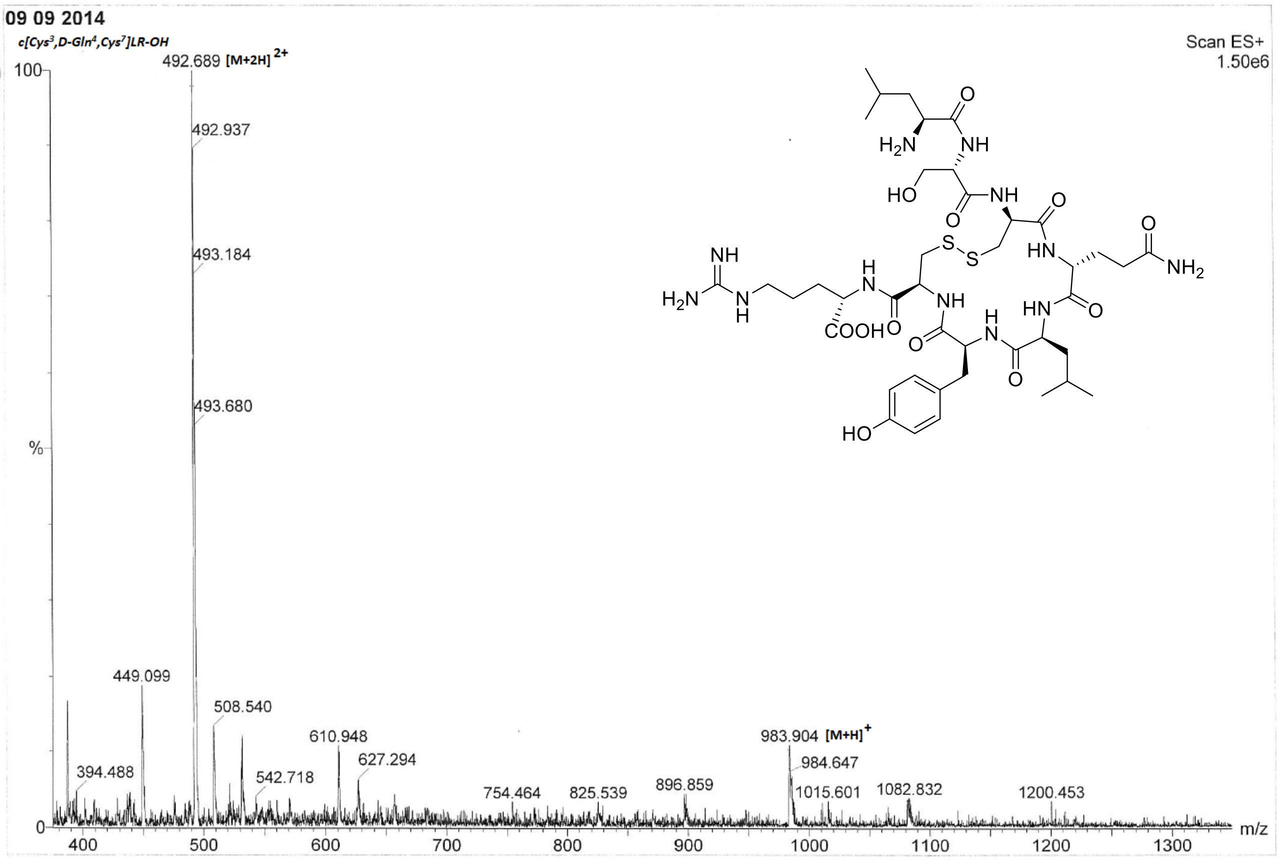

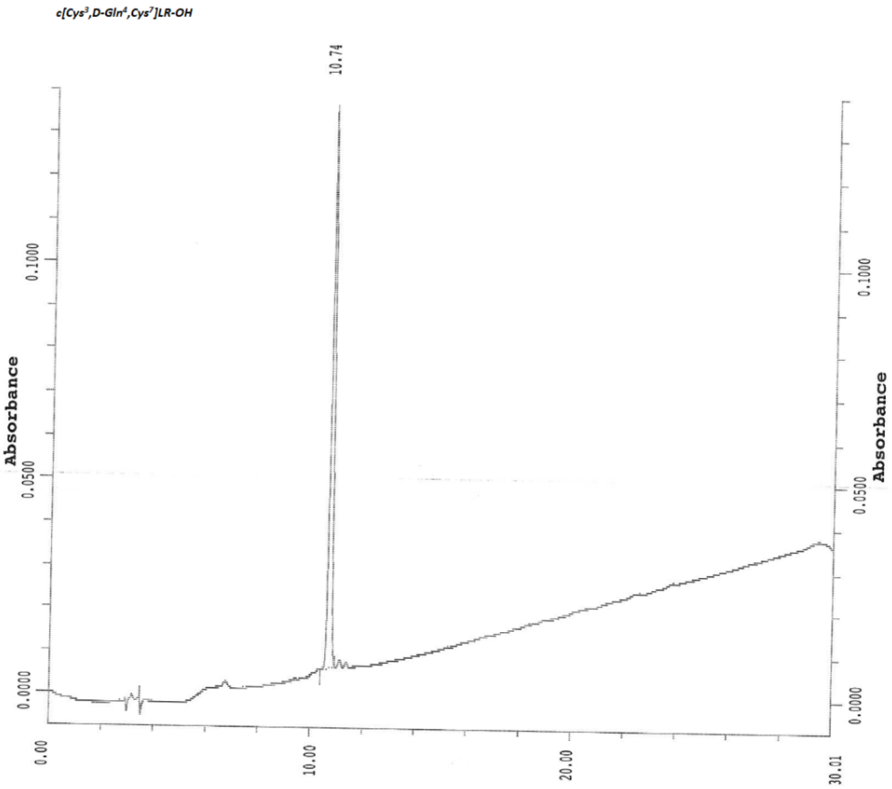

Leu-c[Cys-Pro-Gln-Leu-Cys-]Gln-Arg (6)

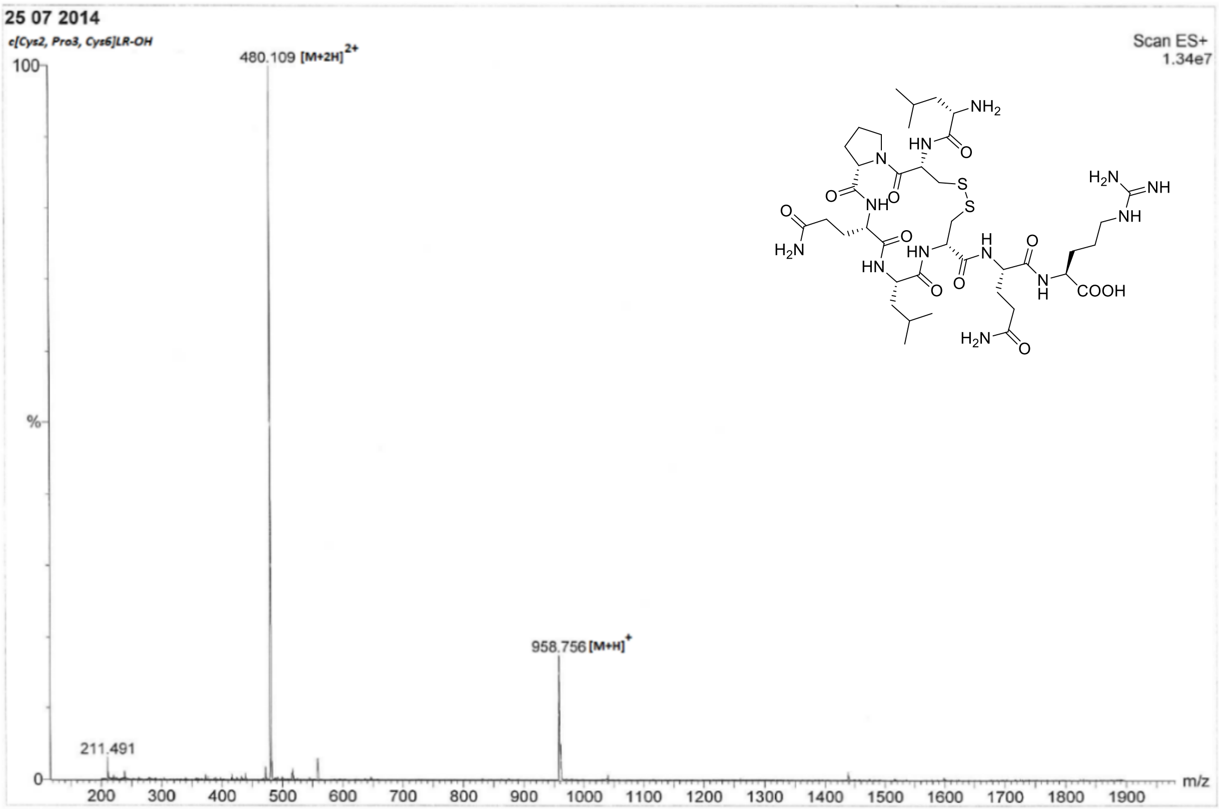

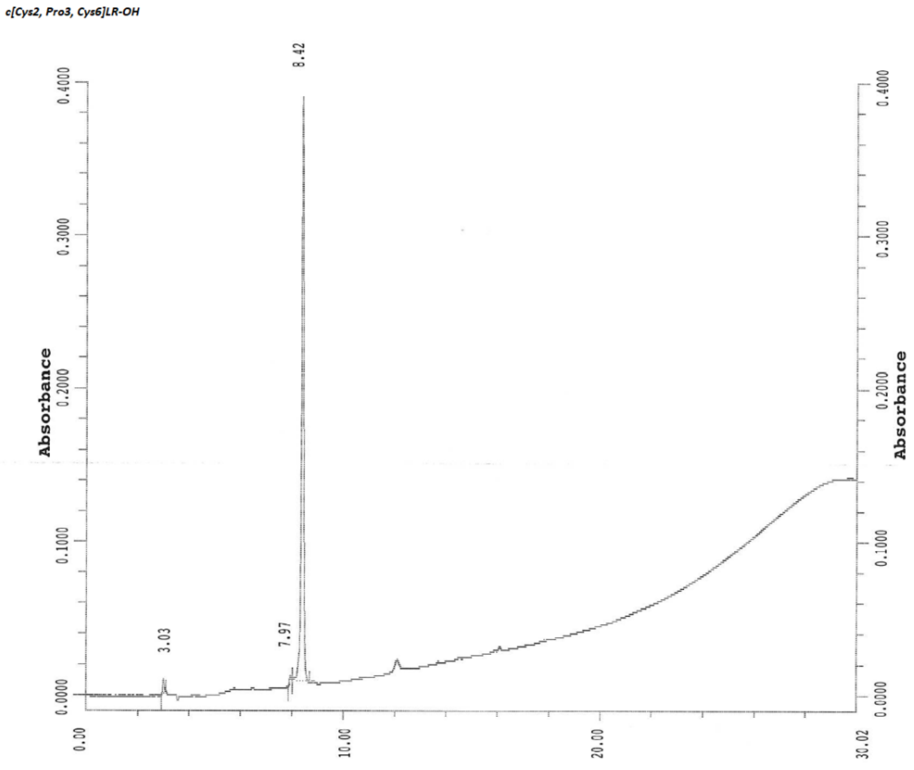

Leu-c[Cys-Pro-Gln-Leu-Tyr-Cys-]Arg (7)

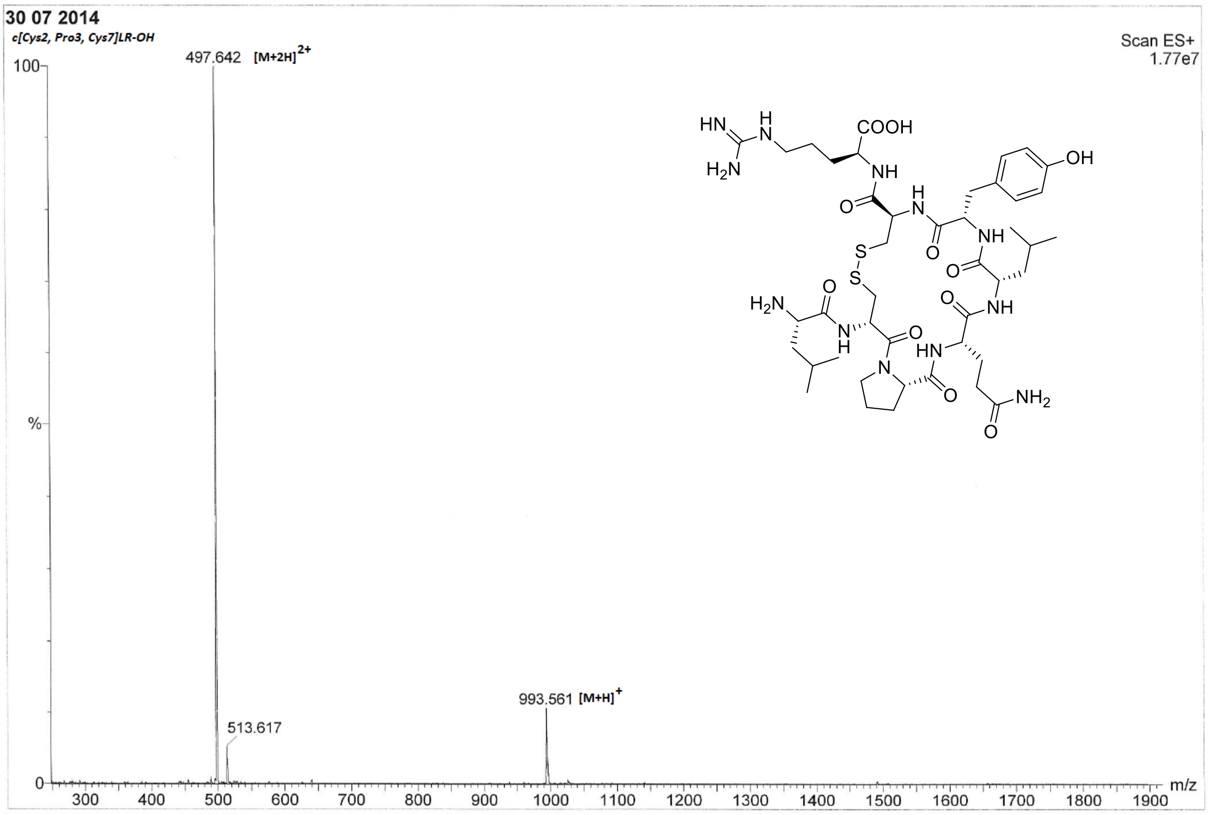

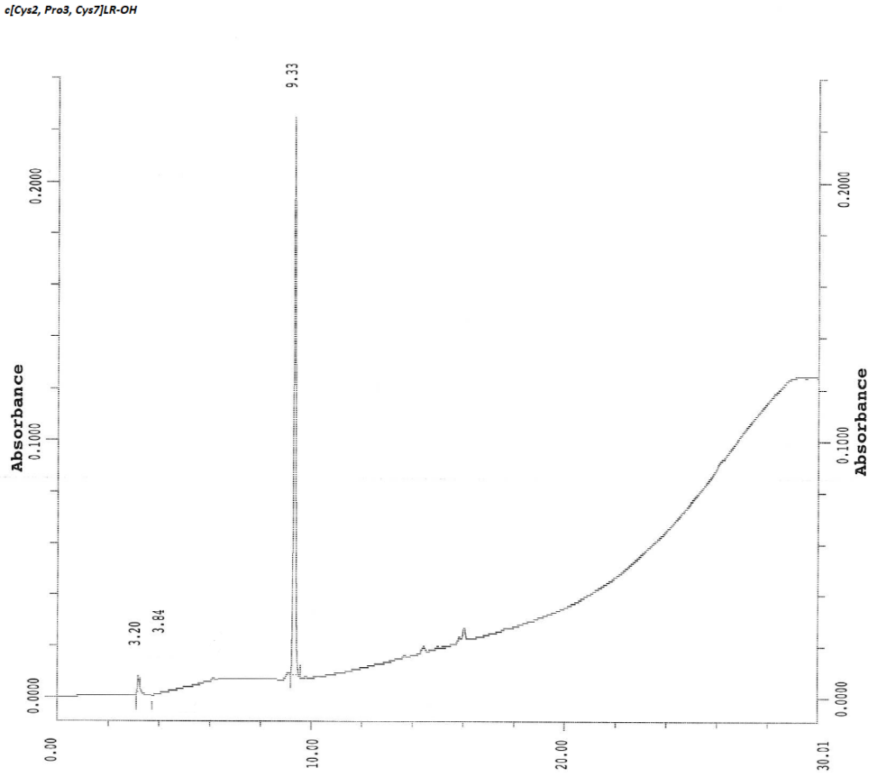

Leu-c[Cys-Pro-Gln-Leu-Tyr-Gln-Cys] (8)

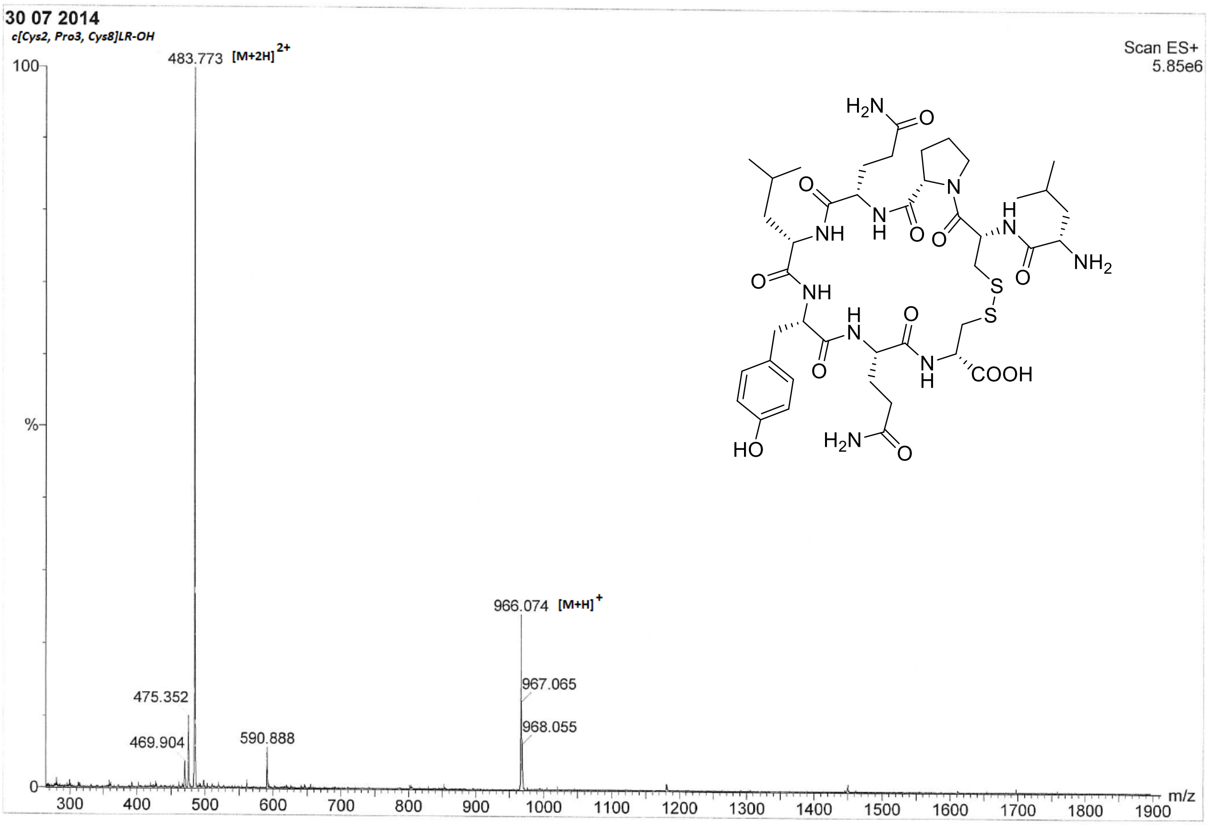

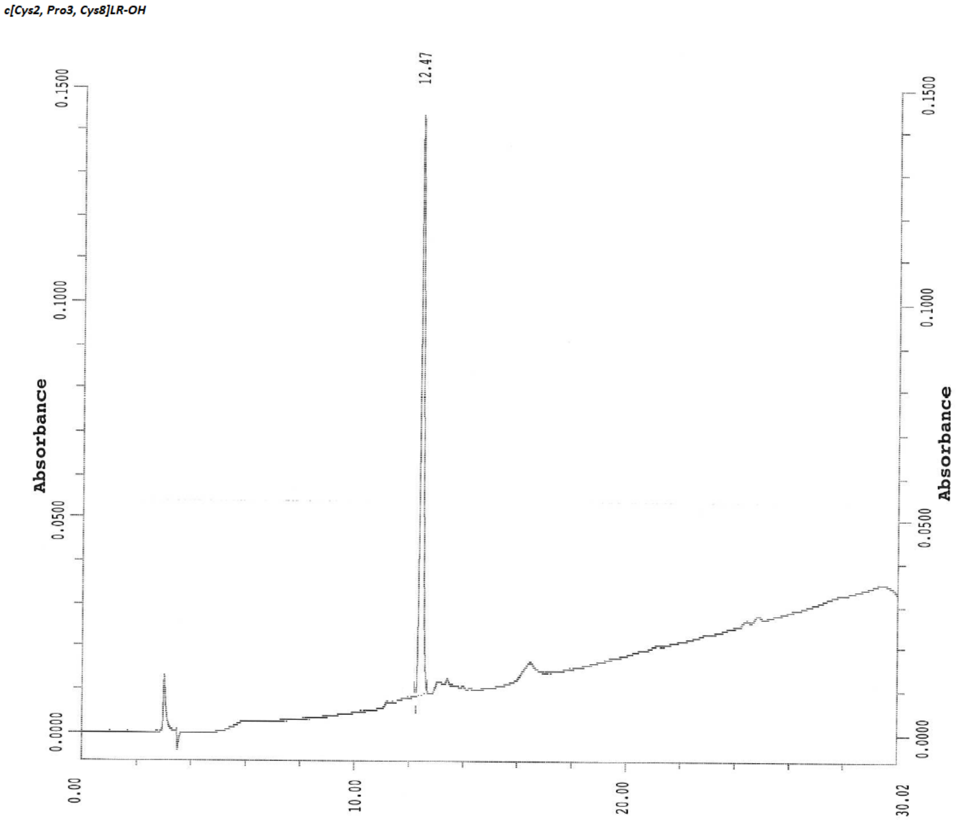

Leu-c[Cys-Pro-Gln-Leu-Tyr-Gln-Arg-CAM] (9)

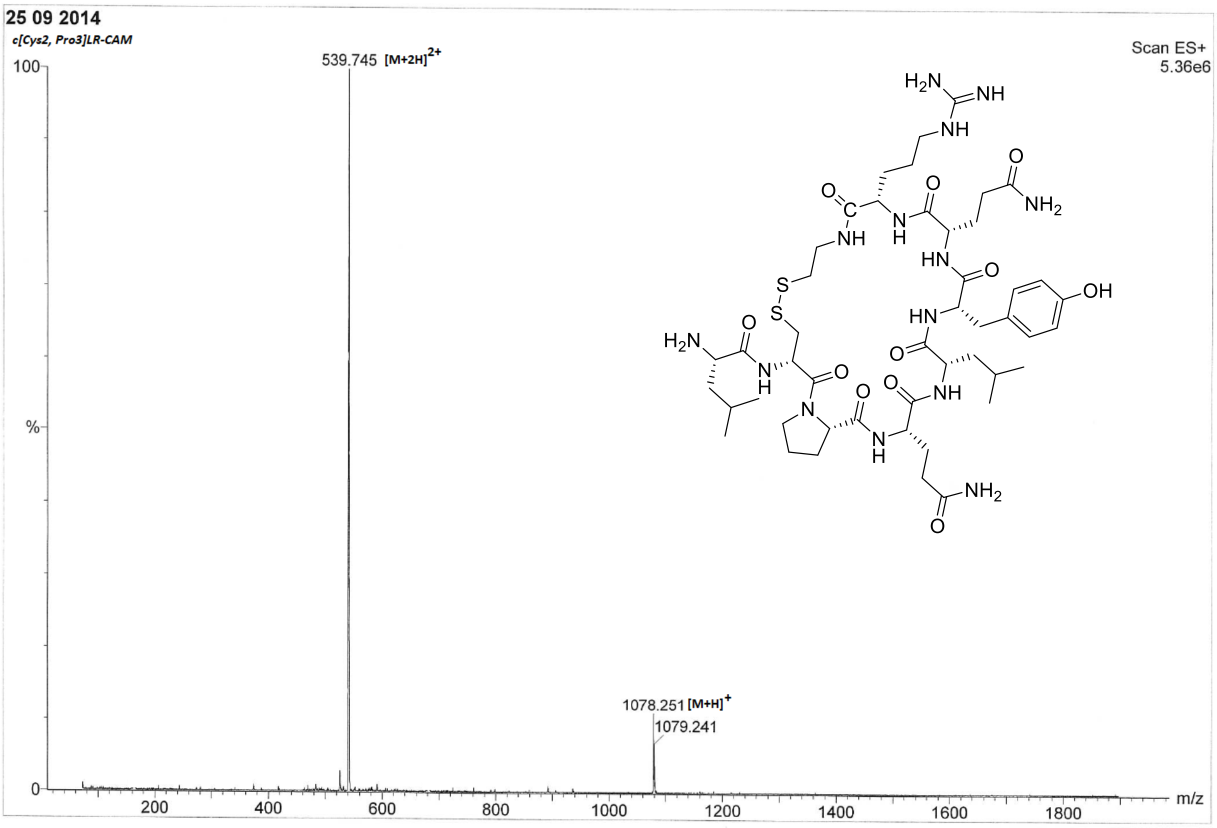

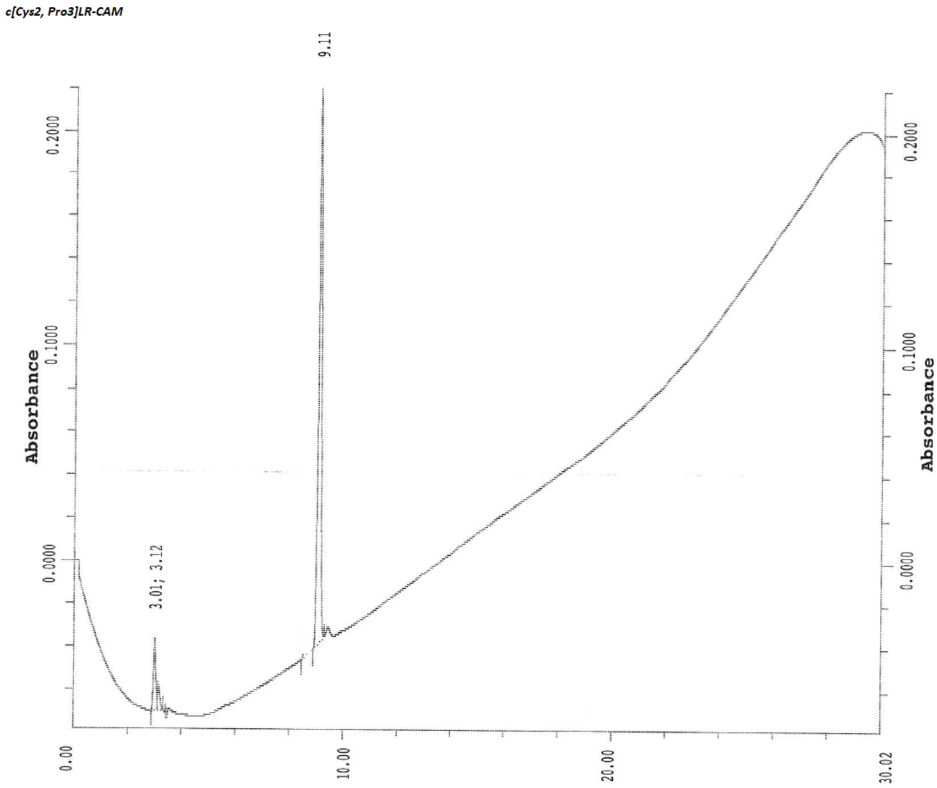

c[Cys-Ser-Pro-Gln-Leu-Tyr-Gln-Cys] (10)

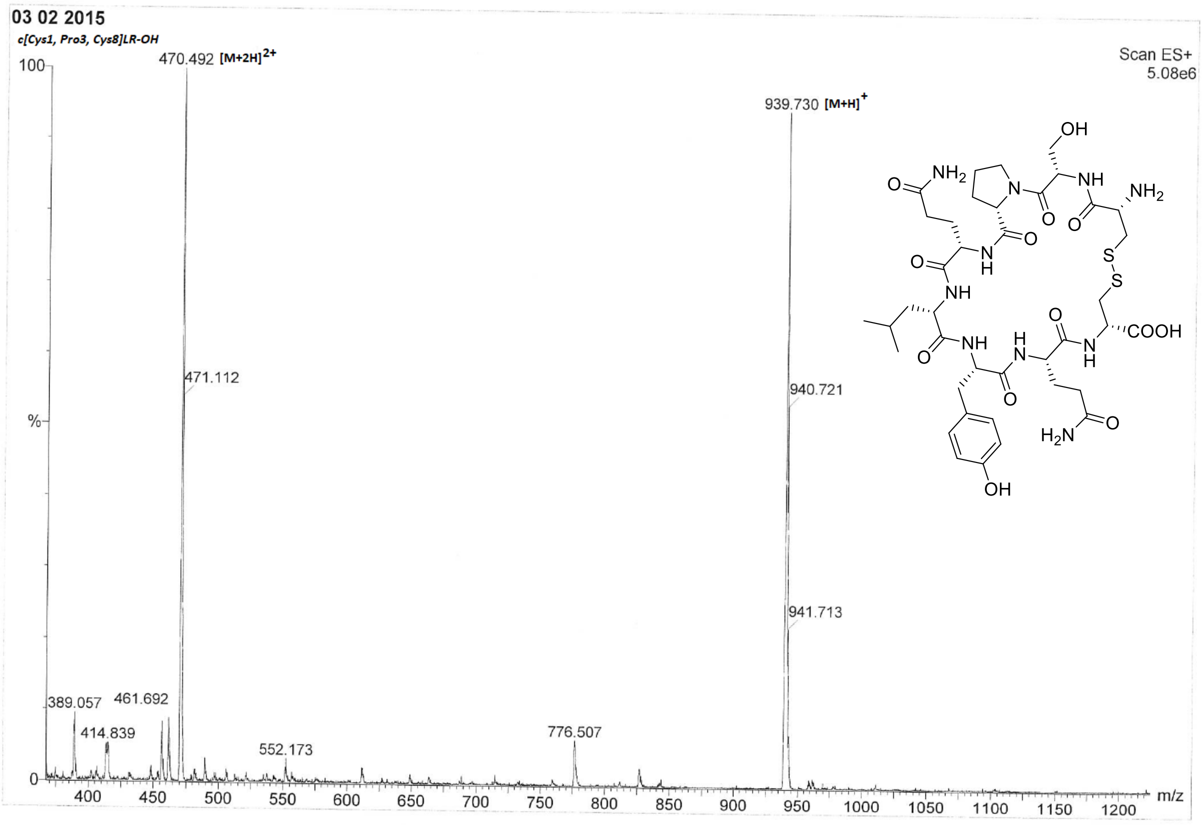

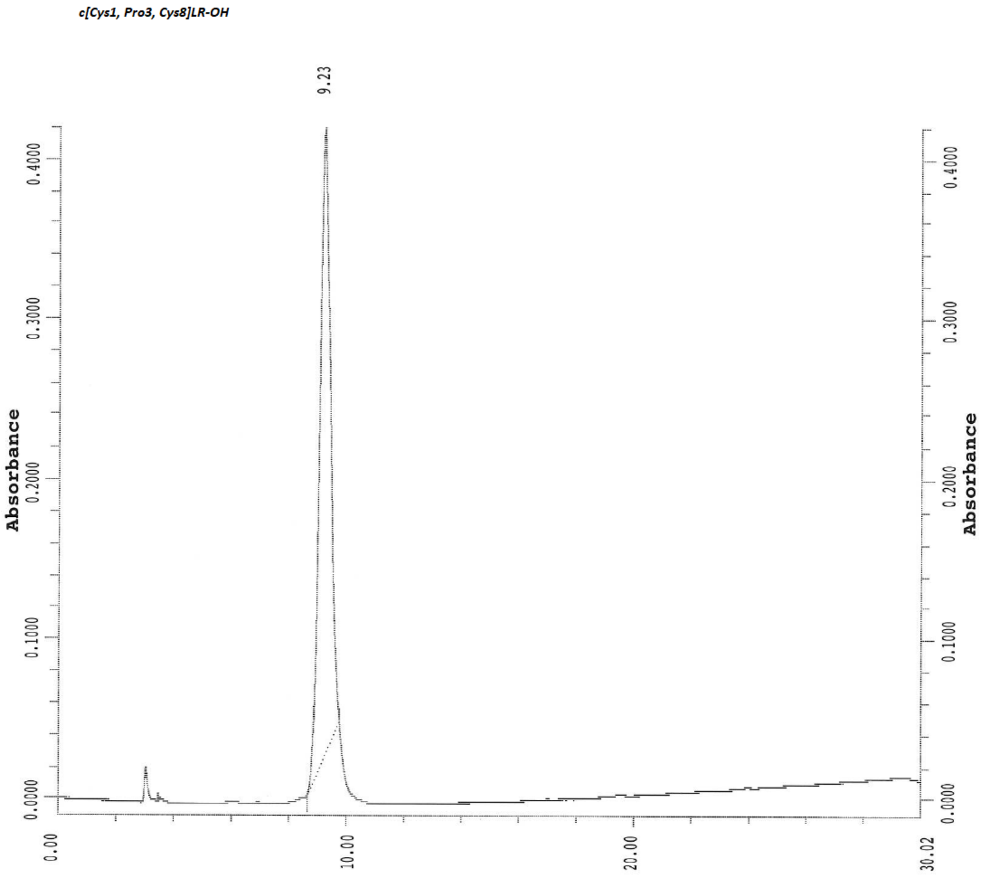

Supplement: Supplementary file 1 [file molecules-24-03493-s001.pdf]
